# Defects in anaplerotic metabolism sensitize *Staphylococcus aureus* small colony variants to bicarbonate

**DOI:** 10.1128/spectrum.01685-25

**Published:** 2025-09-17

**Authors:** Asif Iqbal, Zannatul H. Tumpa, Wyatt W. Wittliff, Bennett J. Blank, Basel H. Abuaita, William N. Beavers

**Affiliations:** 1 Department of Pathobiological Sciences, Louisiana State University and Agricultural and Mechanical College, School of Veterinary Medicine 5779, Baton Rouge, Louisiana, USA; 2 Louisiana State University School of Veterinary Medicine Mass Spectrometry Resource Center, Louisiana State University and Agricultural and Mechanical College, School of Veterinary Medicine, Baton Rouge, Louisiana, USA; University of Florida, Gainesville, Florida, USA

**Keywords:** *Staphylococcus aureus*, bicarbonate, small colony variants, anaplerotic metabolism, pyruvate, oxaloacetate

## Abstract

*Staphylococcus aureus* is a facultative anaerobe that can generate energy through oxidative phosphorylation or solely glycolysis, and inhibiting oxidative phosphorylation results in the formation of small colony variants (SCVs). SCVs lack a proton motive force (PMF), increasing antibiotic tolerance and contributing to persistent infection in the host. Bicarbonate is an abundant antimicrobial compound in the human host that *S. aureus* encounters during infection. Bicarbonate alters the PMF, enhancing antibiotic susceptibility in *S. aureus*, but its impact on *S. aureus* SCVs remains unexplored. We report that bicarbonate inhibits the growth of *S. aureus* SCVs at concentrations that do not affect *S. aureus* wild type, due to defective bicarbonate anaplerotic metabolism, resulting in increased cytoplasmic pH and alkaline toxicity. Inactivation of pyruvate carboxylase (Pyc), a critical enzyme in bicarbonate anaplerotic metabolism that combines bicarbonate and pyruvate to form oxaloacetate, increases bicarbonate sensitivity in *S. aureus*, indicating that bicarbonate anaplerotic metabolism plays a vital role in bicarbonate detoxification. While SCVs upregulate Pyc in response to bicarbonate, cellular pyruvate levels are insufficient to sustain bicarbonate anaplerotic metabolism. Exogenous pyruvate restores bicarbonate anaplerotic metabolism and lowers the cytoplasmic pH, protecting SCVs from bicarbonate toxicity. Cytoplasmic pH alterations by bicarbonate also resensitize SCVs to aminoglycosides. *S. aureus* treated with bicarbonate is more susceptible to neutrophil killing, indicating that bicarbonate decreases the virulence of *S. aureus*. This study identifies bicarbonate anaplerotic metabolism as a *S. aureus* detoxification mechanism for bicarbonate toxicity and demonstrates that modulating anaplerotic metabolism may be an effective treatment for *S. aureus* infections.

**IMPORTANCE:**

*Staphylococcus aureus* is one of the major bacterial contributors to human deaths around the world. Metabolic flexibility allows *S. aureus* to alter energy generation and resist oxidative and antibiotic killing, facilitating persistence in the host. Bicarbonate has been used for over a century for cleaning and hygiene without completely understanding its antimicrobial properties. We report that small colony variants (SCVs) are defective for bicarbonate anaplerotic metabolism, which is required to detoxify bicarbonate. As a result, bicarbonate inhibits the growth of SCVs by alkalinizing the cytoplasm. Cytoplasmic alkalinization also resensitizes SCVs to aminoglycoside killing, implicating bicarbonate as an effective antimicrobial adjuvant for treating glycolytic *S. aureus*. Our study defines the impacts of bicarbonate on the growth of SCVs and the metabolic pathways involved in detoxification, indicating that bicarbonate could be effective at controlling chronic *S. aureus* infections.

## INTRODUCTION

*Staphylococcus aureus* is a leading cause of gram-positive sepsis and death in humans, contributing to more than one million deaths per year globally (1). Nearly 30% of the human population is asymptomatically colonized by *S. aureus* on their skin or in their nares (2), and this pathogen can infect every host tissue, including the eyes, throat, blood, intestine, lungs, heart, and bone marrow (3–10). In response to staphylococcal infections, the host immune system recruits neutrophils to the infection site. Neutrophils kill *S. aureus* through phagocytosis and by releasing cocktails of reactive oxygen species, antimicrobial peptides, and polyunsaturated fatty acids (11–16). However, *S. aureus* can also alter its metabolism, subverting the immune response and antibiotic efficacy, allowing it to escape killing and cause life-threatening chronic infections (17, 18). A subset of this bacterial population that is important in persistent infections is known as small colony variants (SCVs), named for their slow growth rate (19, 20).

SCVs are caused by mutations in genes important for the electron transport chain (ETC) function and are selected by the host immune response or antibiotics (21). The most commonly isolated clinical *S. aureus* SCVs have mutations in the genes encoding enzymes of the menaquinone (e.g.*, menD*) or heme (e.g*., pbgS,* previously named *hemB*) biosynthetic pathways (19, 22, 23). Both menaquinone and heme are essential enzymatic cofactors for the ETC (19, 22). These mutations inactivate oxidative phosphorylation, dissipating the proton motive force (PMF) and forcing *S. aureus* SCVs to rely solely on glycolysis for energy generation. Therefore, SCVs produce lower levels of ATP and grow more slowly. SCVs also produce fewer toxins, making them less virulent but less immunogenic compared to a wild-type strain. The low metabolic activity of SCVs drives increased tolerance to antibiotics and reactive oxygen species and an increased propensity to form biofilms (17, 24, 25). SCVs are considered a reservoir for persistent *S. aureus* infections and are frequently isolated from patients with life-threatening chronic infections, including cystic fibrosis, osteomyelitis, and endocarditis (26–28). Therefore, it is important to define the molecular mechanisms by which the host immune system and current antibiotic treatments fail to clear staphylococcal infections from the host, which will identify therapeutic weaknesses to target in the treatment of persistent *S. aureus* infections.

Bicarbonate is widely used in the dental, food, and agriculture industries for its antimicrobial properties (29–31). In the human host, bicarbonate functions to maintain the optimal pH of blood and tissues (29, 30). The physiological concentration of bicarbonate in the human body varies across tissue types, with the pancreas secreting the highest concentration of bicarbonate at 150 mM, while blood and salivary glands contain 24 and 60 mM bicarbonate, respectively (29). The abundance of bicarbonate in human tissues suggests that bacterial pathogens regularly encounter it during infection and will possess detoxification mechanisms to avoid alkaline toxicity.

The growth of both gram-negative and gram-positive bacteria is inhibited by bicarbonate, including *Escherichia coli*, *S. aureus*, *Streptococcus mutans*, *Pseudomonas aeruginosa*, and *Lactobacillus plantarum* (32, 33). Bicarbonate alters susceptibility to a wide range of antibiotics in *E. coli* and *S. aureus* by perturbing the PMF (31, 34), and inhibits the synthesis of wall teichoic acids, enhancing the susceptibility of β-lactam antibiotics in methicillin-resistant *S. aureus* (MRSA) (35, 36). MpsAB was recently identified as a bicarbonate transporter in *S. aureus,* and deletion of that transporter causes a growth defect, implying that *S. aureus* uses bicarbonate as a carbon source (37). MpsAB is the only dissolved inorganic carbon (DIC) import system defined in *S. aureus* (38, 39), and *S. aureus* does not encode a carbonic anhydrase enzyme that interconverts carbon dioxide and bicarbonate (38, 40). While significant progress has been made in elucidating the sodium bicarbonate transporter and the effects of bicarbonate on antibiotic susceptibility in wild-type *S. aureus,* the impact of bicarbonate on *S. aureus* SCVs remains unexplored.

In this study, we investigated the impact of sodium bicarbonate on the *S. aureus* SCV strains Δ*menD*, *pbgS::Tn*, and *qoxB::Tn* Δ*cydB* (deficient in terminal oxidase of the ETC) in the *S. aureus* USA300 LAC JE2 background (41). USA300 is currently the dominant *S. aureus* clinical strain in the United States (42). Our findings reveal that *S. aureus* SCVs are more sensitive than wild type to bicarbonate due to defects in bicarbonate anaplerotic metabolism. Low levels of pyruvate in SCVs prove insufficient to maintain bicarbonate anaplerotic metabolism, resulting in alkalinization of cytoplasm and toxicity in SCVs following bicarbonate treatment. SCVs are tolerant to aminoglycosides due to their altered PMF (43–45), and we discovered that bicarbonate restores aminoglycoside susceptibility in SCVs. Finally, hypochlorous acid (HOCl) sensitizes *S. aureus* to bicarbonate, enhancing the ability of neutrophils to kill the pathogen and demonstrating cooperativity between blood chemistry and immune system activity. Our study defines bicarbonate sensitivity in *S. aureus* SCVs at the molecular level and indicates that bicarbonate anaplerotic metabolism can be exploited as a potential therapeutic target to control *S. aureus* infection in the host.

## RESULTS

### Bicarbonate inhibits the growth of *S. aureus* SCVs

**Fig 1:**
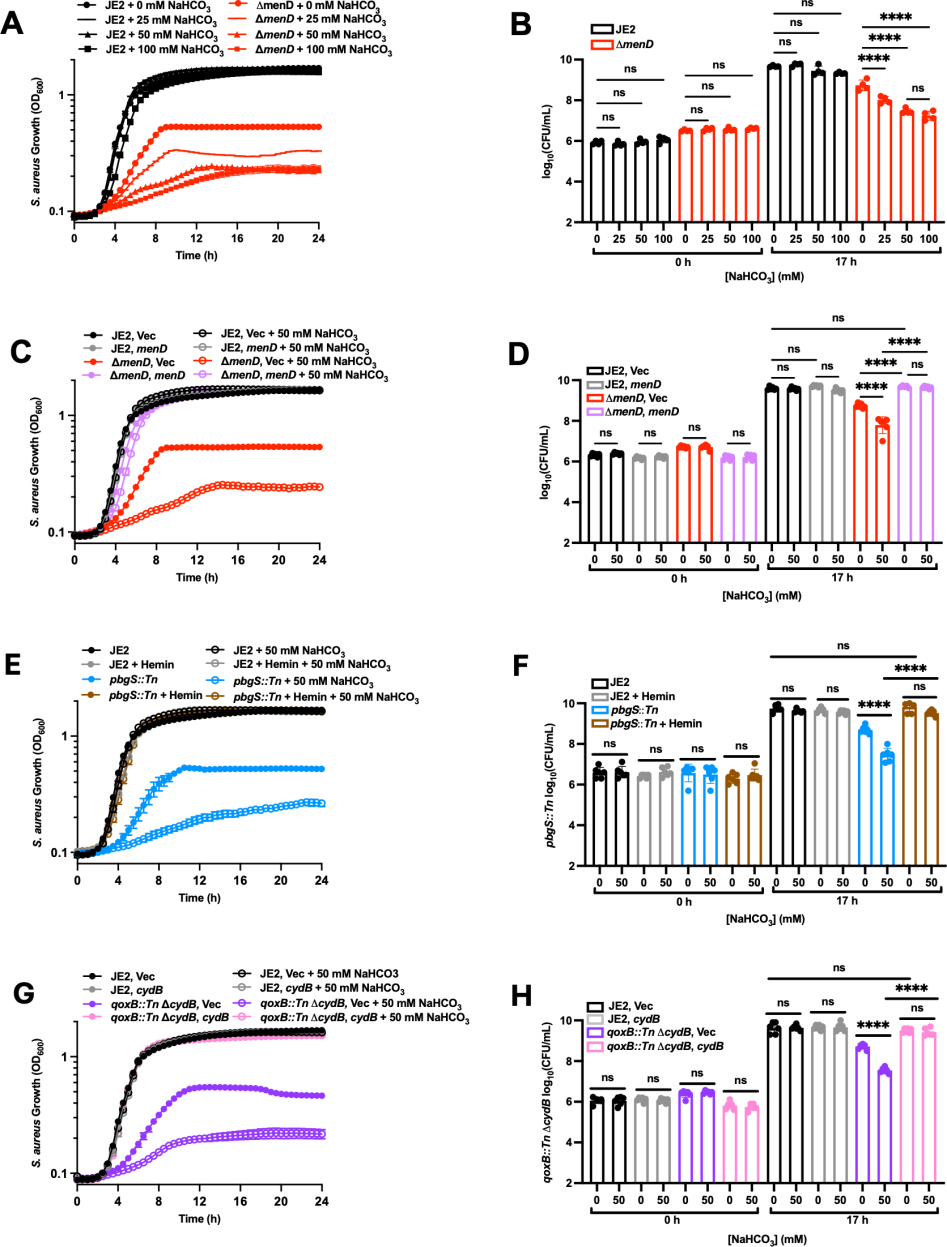
Bicarbonate inhibits the growth of *S. aureus* SCVs. (**A**) The growth of JE2 and Δ*menD* treated with 0, 25, 50, or 100 mM NaHCO_3_ was measured by monitoring the OD_600_ every 30 min for 24 h. Data are presented as mean ± SEM of six biological replicates. (**B**) JE2 and Δ*menD* were grown in 0, 25, 50, and 100 mM NaHCO_3_ for 17 h followed by dilution plating to enumerate the CFUs. Data are presented as mean ± SD of four biological replicates. One-way ANOVA was used to determine statistical significance where ns, *P* > 0.05; ****P < 0.0001. (**C**) The growth of JE2 and Δ*menD* transformed with the empty vector pOS1.P*_Igt_* (Vec) or pOS1.P*_Igt_.menD* (*menD*) ± 50 mM NaHCO_3_ was measured by OD_600_ at 30 min intervals for 24 h. Data are presented as mean ± SEM of six biological replicates. (**D**) JE2 and Δ*menD* were transformed with the empty vector pOS1.P*_Igt_* (Vec) or pOS1.P*_Igt_.menD* (*menD*) ± 50 mM NaHCO_3_ were grown for 17 h followed by dilution plating to enumerate the CFUs. Data are presented as mean ± SD of six biological replicates. One-way ANOVA was used to determine statistical significance where ns, *P* > 0.05; ****P < 0.0001. (**E**) The growth of JE2 and *pbgS::Tn* ± 5 µM hemin chloride ± 50 mM NaHCO_3_ was measured by OD_600_ at 30 min intervals for 24 h. Data are presented as mean ± SEM of six biological replicates. (**F**) JE2 and *pbgS::Tn* ± µM hemin chloride ± 50 mM NaHCO_3_ were grown for 17 h followed by dilution plating to enumerate the CFUs. Data are presented as mean ± SD of six biological replicates. One-way ANOVA was used to determine statistical significance where ns, *P* > 0.05; ****P < 0.0001. (**G**) The growth of JE2 and *qoxB::Tn* Δ*cydB* transformed with empty vector pOS1.P*_Igt_* (Vec) or pOS1.P*_Igt_.cydB* (*cydB*) ± 50 mM NaHCO_3_ was measured by OD_600_ at 30 min intervals for 24 h. Data are presented as mean ± SEM of six biological replicates. (**H**) JE2 and *qoxB::Tn* Δ*cydB* were transformed with the empty vector pOS1.P*_Igt_* (Vec) or pOS1.P*_Igt_.cydB* (*cydB*) ± 50 mM NaHCO_3_ were grown for 17 h followed by dilution plating to enumerate the CFUs. Data are presented as mean ± SD of six biological replicates. One-way ANOVA was used to determine statistical significance where ns, *P* > 0.05; ****P < 0.0001.

To test the effect of bicarbonate on *S. aureus*, we measured the growth of strains JE2 and Δ*menD* by monitoring growth kinetics. Bicarbonate inhibits the growth of Δ*menD* dose-dependently, while none of the tested concentrations of bicarbonate inhibit the growth of JE2 (Fig. 1A). We also tested whether bicarbonate decreases the viable colony-forming units (CFUs) via dilution plating. Bicarbonate dose-dependently decreases the CFU levels of Δ*menD* compared to untreated Δ*menD*, culminating in a 16-fold decrease in CFUs following 100 mM bicarbonate treatment (Fig. 1B). No concentration of bicarbonate tested affects the JE2 CFUs (Fig. 1B). Bicarbonate sensitivity is complemented *in trans* in the Δ*menD* strain by constitutively expressing *menD* from the pOS1 plasmid, confirming defects in menaquinone biosynthesis as the cause of bicarbonate sensitivity (Fig. 1C and D). These results indicate that Δ*menD* is more sensitive to bicarbonate compared to the wild-type strain. To determine whether bicarbonate sensitivity is a phenotype specific for Δ*menD*, we tested bicarbonate sensitivity in the SCV strain *pbgS::Tn*. We found that *pbgS::Tn* is also highly sensitive to bicarbonate compared to JE2 (Fig. 1E and F), and bicarbonate sensitivity in *pbgS::Tn* is chemically complemented by the addition of exogenous heme (Fig. 1E and F). Menaquinone and heme are enzymatic cofactors that may affect other cellular processes in *S. aureus*; therefore, we tested bicarbonate sensitivity in *qoxB::Tn* Δ*cydB* to investigate whether ETC-specific defective *S. aureus* SCVs are similarly sensitive to bicarbonate. We found that *qoxB::Tn* Δ*cydB* is more sensitive to bicarbonate than wild type (Fig. 1G and H), and that bicarbonate sensitivity is complemented by constitutively expressing *cydB in trans* from the pOS1 plasmid (Fig. 1G and H). These data demonstrate that the altered metabolism of *S. aureus* SCVs sensitizes them to bicarbonate.

### Anaerobic conditions sensitize *S. aureus* JE2 to bicarbonate

**Fig 2:**
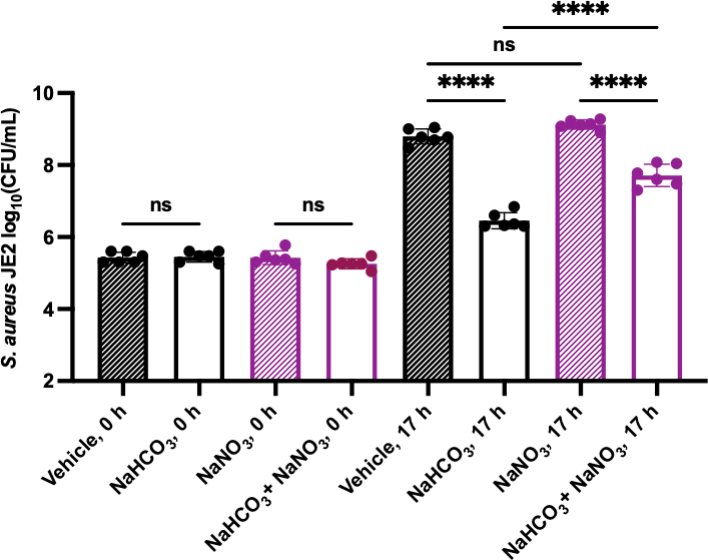
*S. aureus* JE2 becomes sensitive to bicarbonate under anaerobic conditions. In an anaerobic chamber, JE2 was grown for 17 h ± 100 mM NaHCO_3_ ± 100 mM NaNO_3_ followed by dilution plating to enumerate the CFUs. Data are presented as mean ± SD of six biological replicates. One-way ANOVA was used to determine statistical significance where ns, *P* > 0.05; ****P < 0.0001.

*S. aureus* SCVs do not run the tricarboxylic acid (TCA) cycle or oxidative phosphorylation due to inactivating mutations affecting the ETC (46). Similarly, under anaerobic conditions, *S. aureus* wild type does not run oxidative phosphorylation due to the absence of oxygen, the canonical terminal electron acceptor of the ETC (47). Therefore, we tested the effects of bicarbonate under anaerobic conditions and discovered that bicarbonate decreases the CFUs of JE2 by 25-fold compared to untreated (Fig. 2). A higher concentration of bicarbonate was needed to kill *S. aureus* anaerobically, indicating that it does not have a complete SCV phenotype. Anaerobically, *S. aureus* expresses an alternative terminal oxidase (NarGHIJ) of the ETC that uses nitrate as a terminal electron acceptor, facilitating anaerobic respiration (47). Additionally, *S. aureus* can generate nitrate to facilitate anaerobic respiration (48), which leads to the hypothesis that anaerobic respiration provides low levels of resistance to bicarbonate anaerobically. We tested this hypothesis by adding exogenous nitrate that partially rescues the bicarbonate sensitivity of JE2 under anaerobic conditions (Fig. 2). These results demonstrate that bicarbonate sensitivity is inversely correlated with ETC function in *S. aureus*, both aerobically and anaerobically.

### *S. aureus* SCVs are defective for bicarbonate anaplerotic metabolism

**Fig 3:**
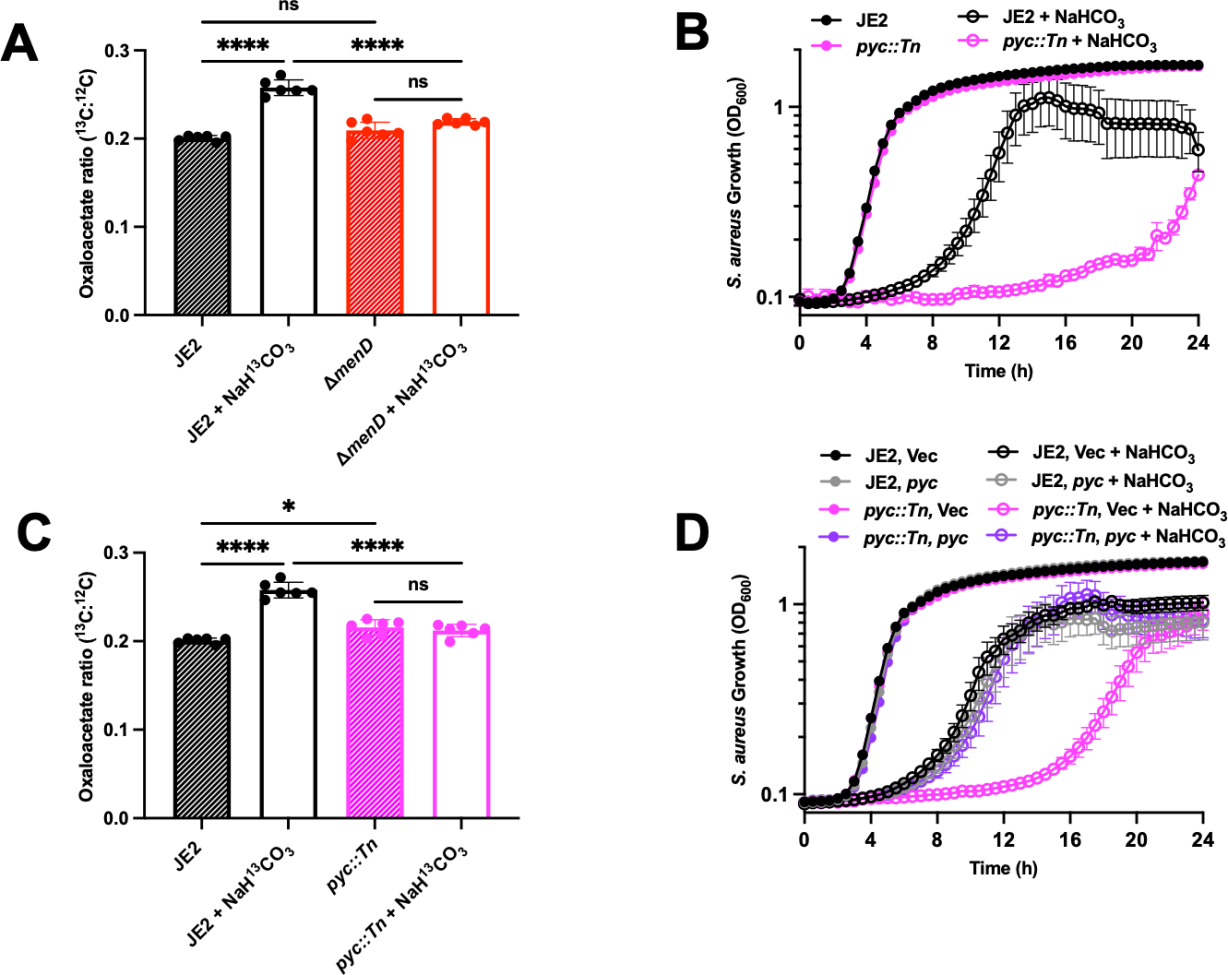
*S. aureus* Δ*menD* is defective in bicarbonate anaplerotic metabolism. (**A**) JE2 and Δ*menD* strains were grown ± 50 mM NaH^13^CO_3_. Following 5 h of growth, oxaloacetate was extracted from the bacterial cells, derivatized, and quantified by LC-MS/MS. Data are presented as mean ± SD of six biological replicates. One-way ANOVA was used to determine statistical significance where ns, *P* > 0.05; ****P < 0.0001. (**B**) The growth of JE2 and *pyc::Tn* ± 200 mM NaHCO_3_ was measured by monitoring the OD_600_ every 30 min for 24 h. Data are presented as mean ± SEM of four biological replicates. (**C**) JE2 and *pyc::Tn* strains were grown ± 50 mM NaH^13^CO_3_. Following 5 h of growth, oxaloacetate was extracted from the bacterial cells, derivatized, and analyzed by LC-MS/MS. Data are presented as mean ± SD of six biological replicates. One-way ANOVA was used to determine statistical significance where ns, *P* > 0.05; ****P < 0.0001. (**D**) The growth of JE2 and *pyc::Tn* was transformed with the control vector pOS1.P*_Igt_* (Vec) or pOS1.P*_Igt_.pyc* (*pyc*) ± 200 mM NaHCO_3_ was measured by monitoring the OD_600_ at 30 min intervals for 24 h. Data are presented as mean ± SEM of six biological replicates.

The bicarbonate anaplerotic reaction consists of the conversion of pyruvate and bicarbonate into oxaloacetate through the enzymatic activity of pyruvate carboxylase (Pyc) (39, 49). We hypothesized that bicarbonate anaplerotic metabolism is a detoxification mechanism in *S. aureus*, and that defects in the reaction will increase bicarbonate toxicity. We quantified bicarbonate anaplerotic metabolism in SCVs by liquid chromatography tandem mass spectrometry (LC-MS/MS) through the production of ^13^C-oxaloacetate from ^13^C-sodium bicarbonate (NaH^13^CO_3_). ^13^C-oxaloacetate increases in JE2 by 28% while no increase in ^13^C-oxaloacetate is observed in Δ*menD* treated with NaH^13^CO_3_ (Fig. 3A). This is likely an underestimation of the flux through the bicarbonate anaplerotic reaction because oxaloacetate is readily converted to other small molecule metabolites, diluting the ^13^C signal. These results suggest that SCVs have defective bicarbonate anaplerotic metabolism, making them sensitive to bicarbonate.

Pyc uses bicarbonate as a substrate to convert pyruvate into oxaloacetate during bicarbonate anaplerotic metabolism (39, 49). We tested the hypothesis that inactivation of *pyc* will sensitize *S. aureus* to bicarbonate and observed that bicarbonate sensitivity increases in *pyc::Tn*. This phenotype is only present at high bicarbonate concentrations (Fig. 3B; Fig. S1), suggesting that additional metabolic pathways may contribute to bicarbonate resistance in *S. aureus*. When *pyc* is inactivated, there is no incorporation of NaH^13^CO_3_ into ^13^C-oxaloacetate, indicating a defective bicarbonate anaplerotic metabolism in *pyc::Tn* (Fig. 3C). The bicarbonate sensitivity of *pyc::Tn* is complemented by constitutive expression of *pyc in trans* from the pOS1 plasmid (Fig. 3D). These data demonstrate that Pyc drives bicarbonate anaplerotic metabolism in *S. aureus*, and that this metabolism protects *S. aureus* from bicarbonate toxicity.

### Pyruvate deficiency deprives the bicarbonate anaplerotic reaction of an essential substrate in *S. aureus* SCVs

**Fig 4:**
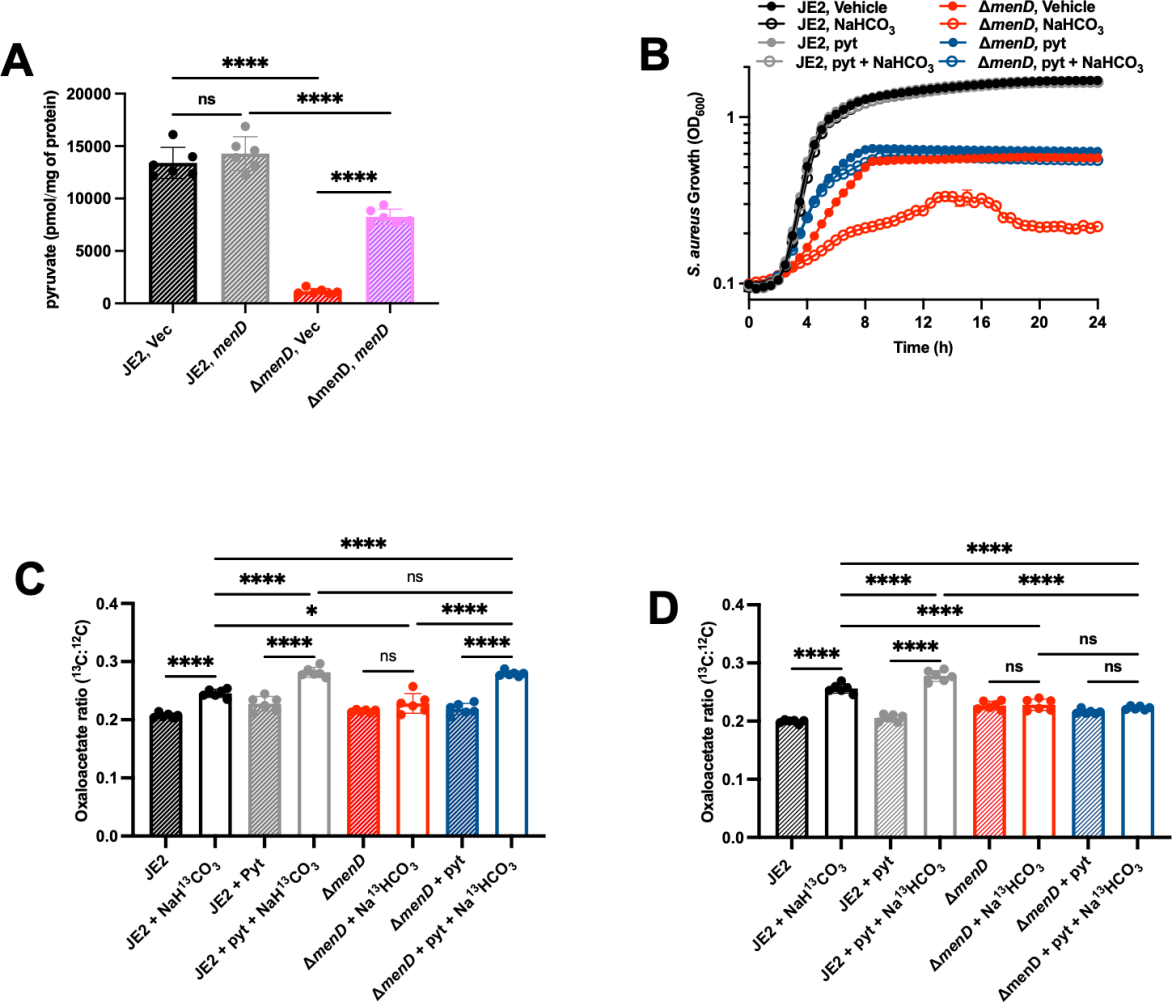
Pyruvate supplementation restores bicarbonate anaplerotic metabolism, protecting *S. aureus* Δ*menD* from bicarbonate toxicity. (**A**) JE2 and Δ*menD* were transformed with the control vector pOS1.P*_Igt_* (Vec) or pOS1.P*_Igt_.menD* (*menD*) and were grown for 5 h before pyruvate was extracted from the bacterial cells, derivatized, and quantified by LC-MS/MS. Data are presented as mean ± SD of six biological replicates. One-way ANOVA was used to determine statistical significance where ns, *P* > 0.05; ****P < 0.0001. (**B**) The growth of *S. aureus* JE2 and Δ*menD* was measured by monitoring the OD_600_ at 30 min intervals for 24 h ± 50 mM NaHCO_3_ ± 4.5 mM pyruvate (pyt). Data are presented as mean ± SEM of three biological replicates. (**C**) JE2 and Δ*menD* cultures were grown ± 50 mM NaH^13^CO_3_ ± 18 mM pyruvate. Following 2.5 h of growth, oxaloacetate was extracted from the bacterial cells, derivatized, and quantified by LC-MS/MS. Data are presented as mean ± SD of six biological replicates. One-way ANOVA was used to determine statistical significance where ns, *P* > 0.05; *P < 0.05; ****P < 0.0001. (**D**) JE2 and Δ*menD* cultures were grown ± 50 mM NaH^13^CO_3_ ± 18 mM pyruvate. Following 5 h of growth, oxaloacetate was extracted from the bacterial cells, derivatized, and quantified by LC-MS/MS. Data are presented as mean ± SD of six biological replicates. One-way ANOVA was used to determine statistical significance where ns, *P* > 0.05; ****P < 0.0001.

Proteomic analyses demonstrate that bicarbonate induces Pyc expression in *S. aureus* Δ*menD* (2.1 fold change, adjusted *P* = 0.00027), but not in JE2 (1.0 fold change, adjusted *P* = 0.83). These data demonstrate that the defect in bicarbonate anaplerotic metabolism observed in Δ*menD* is not due to decreased Pyc expression and that the increased expression of Pyc may be a bicarbonate stress response. With bicarbonate and Pyc levels sufficient to support anaplerotic metabolism, we quantified pyruvate levels in JE2 and Δ*menD*. Pyruvate levels decrease by 11-fold in Δ*menD* compared to JE2 and are partially complemented by the expression of *menD in trans* (Fig. 4A). Exogenous pyruvate increases the growth rate of Δ*menD* and fully protects the strain from bicarbonate toxicity (Fig. 4B). Exogenous pyruvate does not affect the growth of JE2 in any of the tested conditions, presumably because the wild-type strain is pyruvate replete (Fig. 4B). Increased growth rates and protection from bicarbonate toxicity were also observed in *pbgS::Tn* and *qoxB::Tn* Δ*cydB* following the addition of exogenous pyruvate, indicating a common mechanism of defective bicarbonate anaplerotic metabolism in SCVs (Fig. S2).

Exogenous pyruvate combined with NaH^13^CO_3_ increases ^13^C-oxaloacetate in both JE2 and Δ*menD* following 2.5 h of growth (Fig. 4C). Δ*menD* grown with the combination of pyruvate and NaH^13^CO_3_ for 2.5 h produces significantly higher levels of ^13^C-oxaloacetate compared to JE2 grown with only NaH^13^CO_3_, presumably due to excess pyruvate and the increased Pyc expression in Δ*menD* (Fig. 4C). Following 5 h of growth, only JE2 has increased levels of ^13^C-oxaloacetate (Fig. 4D). All growth conditions of Δ*menD* have similar levels of ^13^C-oxaloacetate at 5 h, implying that the exogenous pyruvate is completely consumed by 5 h post-treatment (Fig. 4D). These data indicate that whether pyruvate is plentiful, bicarbonate can be metabolized into oxaloacetate in SCVs. The return to basal oxaloacetate levels in Δ*menD* at 5 h post-treatment indicates that oxaloacetate is rapidly used for downstream metabolic processes (Fig. 4C and D). These findings demonstrate that decreased pyruvate levels explain the defective bicarbonate anaplerotic metabolism and increased bicarbonate sensitivity in SCVs.

### Bicarbonate increases the cytoplasmic pH of *S. aureus,* resulting in alkaline toxicity

**Fig 5:**
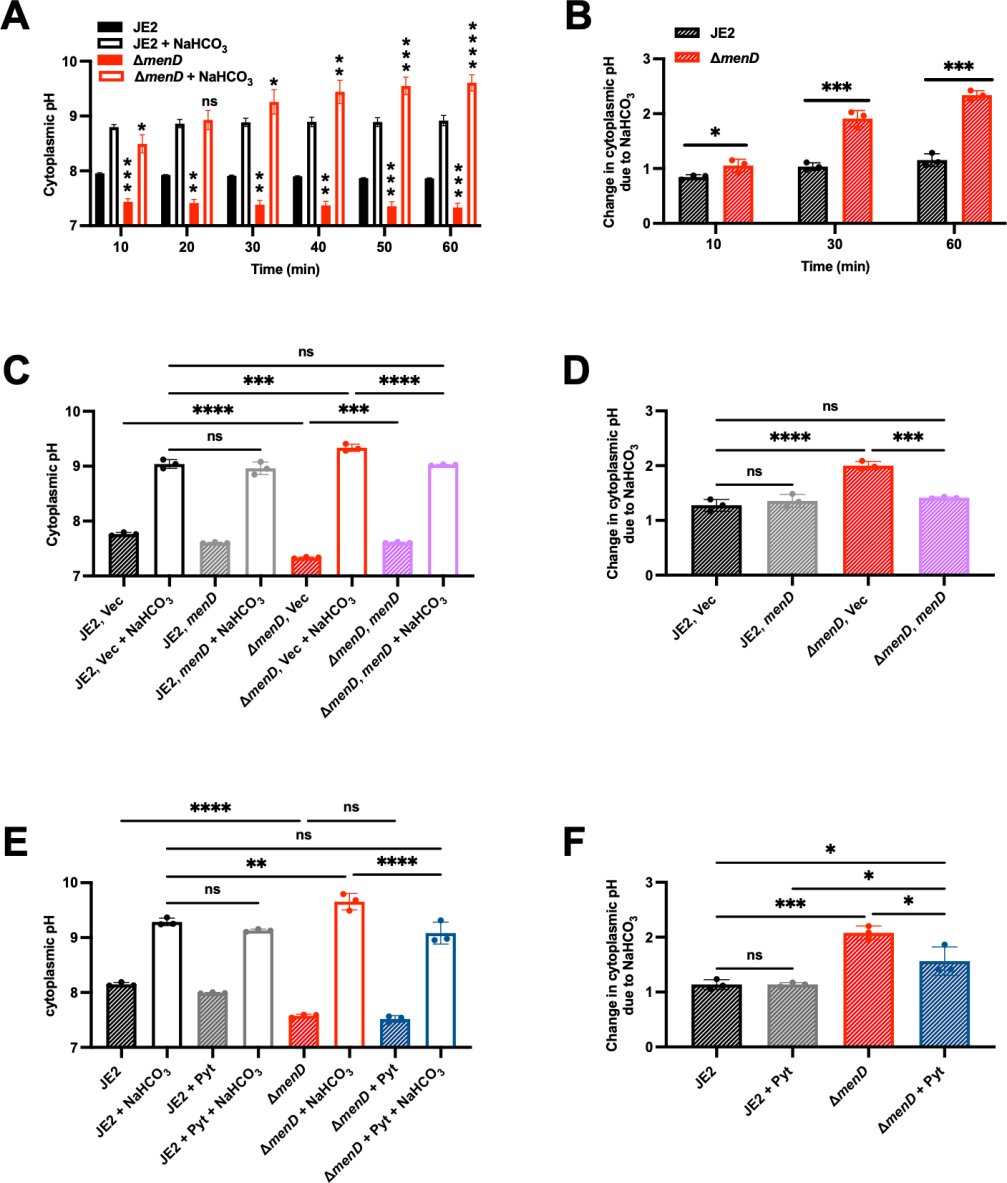
Defective bicarbonate anaplerotic metabolism results in increased cytoplasmic alkalinization in bicarbonate-treated *S. aureus* Δ*menD*. (**A**) The cytoplasmic pH of JE2 and Δ*menD* ± 25 mM NaHCO_3_ was measured using BCECF-AM dye. Data are presented as mean ± SD of three biological replicates. One-way ANOVA was used to determine statistical significance. The statistical comparisons shown compare JE2 to Δ*menD* (closed bars) and JE2 + bicarbonate to △*menD* + bicarbonate (open bars) at each time point where ns, *P* > 0.05; *P < 0.05; **P < 0.01; ***P < 0.001; ****P < 0.0001. (**B**) The change in cytoplasmic pH from panel A was calculated for each strain at different time points to determine how the pH changes following 25 mM NaHCO_3_ treatment. Data are presented as mean ± SD of three biological replicates. One-way ANOVA was used to determine statistical significance where *P < 0.05; ***P < 0.001. (**C**) The cytoplasmic pH of JE2 and Δ*menD* was transformed with the empty control vector pOS1.P*_Igt_* (Vec) or pOS1.P*_Igt_.menD* (*menD*) ± 25 mM NaHCO_3_ was measured following 60 min of growth using BCECF-AM dye. Data are presented as mean ± SD of three biological replicates. One-way ANOVA was used to determine statistical significance where ns, *P* > 0.05; ***P < 0.001; ****P < 0.0001. (**D**) The change in cytoplasmic pH from panel C was calculated for each strain to determine how the pH changes at each time point following 25 mM NaHCO_3_ treatment. Data are presented as mean ± SD of three biological replicates. One-way ANOVA was used to determine statistical significance where ns, *P* > 0.05; ***P < 0.001; ****P < 0.0001. (**E**) The cytoplasmic pH of JE2 and Δ*menD* ± 25 mM NaHCO_3_ ± 18 mM sodium pyruvate (pyt) was measured following 60 min of growth using BCECF-AM dye. Data are presented as mean ± SD of three biological replicates. One-way ANOVA was used to determine statistical significance where ns, *P* > 0.05; **P < 0.01; ****P < 0.0001. (**F**) The change in cytoplasmic pH from panel E was calculated for each strain to determine how the pH changes following treatment ± 25 mM NaHCO_3_ ± 18 mM pyruvate. Data are presented as mean ± SD of three biological replicates. One-way ANOVA was used to determine statistical significance where ns, *P* > 0.05; *P < 0.05; ***P < 0.001.

We tested the hypothesis that defective bicarbonate anaplerotic metabolism results in alkaline stress by measuring the intracellular pH of *S. aureus* strains treated with and without bicarbonate. The basal cytoplasmic pH of Δ*menD* is significantly lower than that of JE2 due to a defective ETC (Fig. 5A). These results suggest that SCVs have decreased levels of intercellular pH because of high levels of lactate produced from the pyruvate generated during glycolysis (50), which is essential for maintaining redox balance and regenerating NAD^+^ (47, 51). The cytoplasmic pH of Δ*menD* rapidly increases following bicarbonate treatment, then continues to increase further during the hour of observation (Fig. 5A). In contrast, the cytoplasmic pH of JE2 also increases rapidly upon treatment but quickly reaches a steady state due to the functional bicarbonate anaplerotic reaction (Fig. 5A). Bicarbonate treatment increases the cytoplasmic pH of Δ*menD* by two pH units, while the cytoplasmic pH of JE2 increases by less than one pH unit compared to basal conditions (Fig. 5B). The expression of *menD in trans* complements the observed pH changes (Fig. 5C and D). Similar cytoplasmic pH changes are observed following bicarbonate treatment in the SCV strains *pbgS::Tn* and *qoxB::Tn* Δ*cydB,* and both phenotypes are complemented by exogenous hemin and the expression of *cydB in trans*, respectively (Fig. S3 and S4).

Pyruvate protects against bicarbonate toxicity in SCVs by supplying ample substrate to drive the bicarbonate anaplerotic reaction (Fig. 4; Fig. S2). We tested the effect of pyruvate on cytoplasmic pH in Δ*menD* treated with bicarbonate, discovering that exogenous pyruvate decreases the cytoplasmic pH by restoring bicarbonate anaplerotic metabolism (Fig. 5E and F). Pyruvate does not alter the cytoplasmic pH of JE2 treated with bicarbonate because pyruvate is already present in excess in *S. aureus* wild type (Fig. 5E and F). These results demonstrate that bicarbonate anaplerotic metabolism protects *S. aureus* from alkaline toxicity induced by bicarbonate.

### Bicarbonate resensitizes *S. aureus* SCVs to aminoglycoside antibiotics

Cytoplasmic alkalinization alters the PMF and increases aminoglycoside susceptibility in both *S. aureus* and *E. coli* (31, 52). Therefore, we hypothesized that bicarbonate would restore aminoglycoside susceptibility to *S. aureus* SCVs. The gentamicin minimum inhibitory concentration (MIC) of JE2 is 0.75 µg/mL and 0.5 µg/mL with and without 25 mM sodium bicarbonate, respectively (Fig. S5), indicating that bicarbonate decreases the gentamicin MIC of JE2 by 1.5-fold. The gentamicin MIC of Δ*menD* is 16 µg/mL and 8 µg/mL with and without 25 mM sodium bicarbonate, respectively (Fig. S5), indicating that bicarbonate decreases the gentamicin MIC of Δ*menD* by twofold. The MIC values of *pbgS::Tn* and *qoxB::Tn* Δ*cydB* strain are also decreased by twofold following bicarbonate treatment (Fig. S5). Previously, it was also reported that the bicarbonate decreases the gentamicin MIC in *S. aureus* Newman strain by twofold (31).

**Fig 6:**
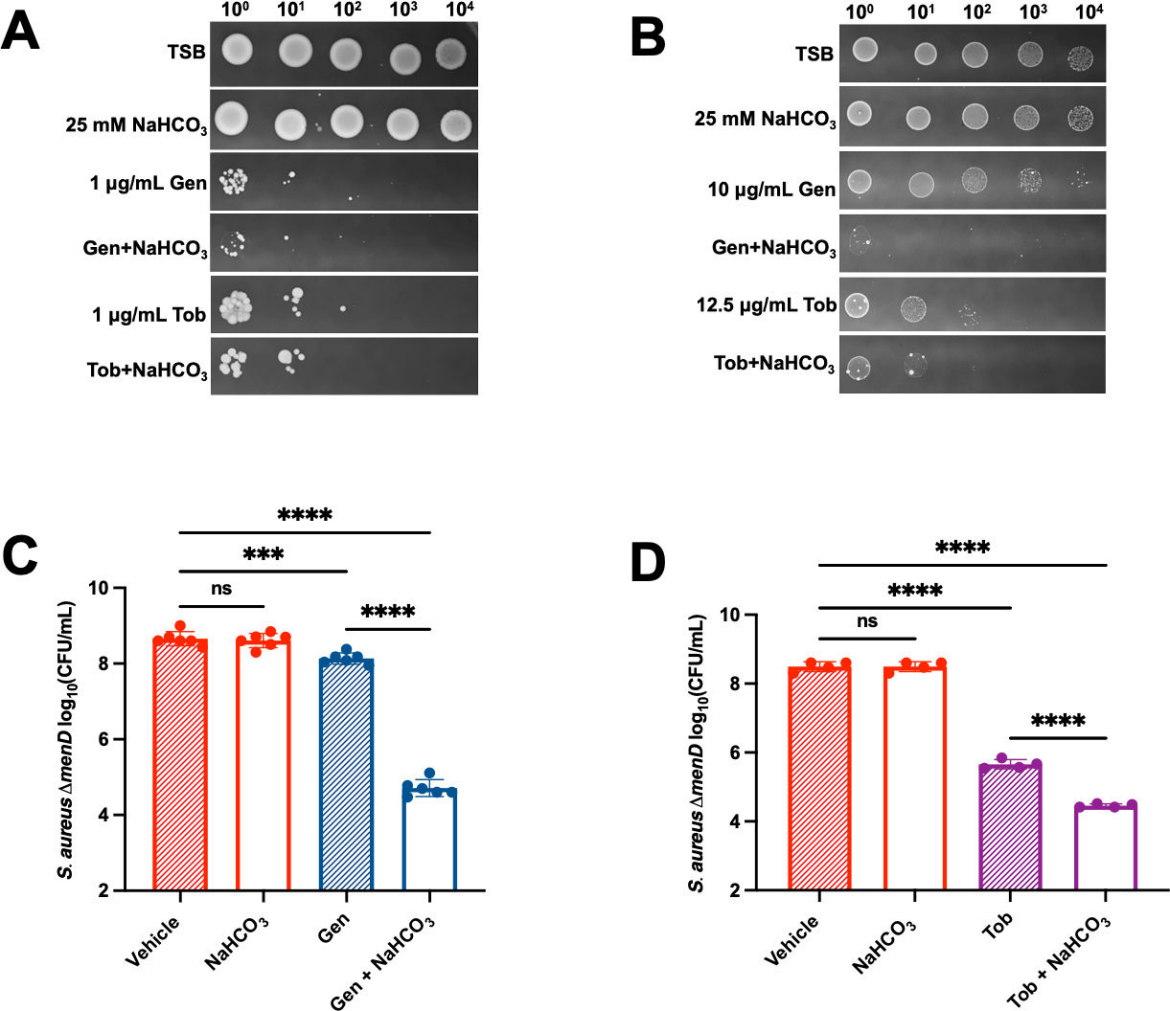
Bicarbonate restores aminoglycoside sensitivity in *S. aureus* △*menD*. Serially diluted JE2 (**A**) and Δ*menD* (**B**) were spotted onto TSA +14 mM glucose ± 25 mM NaHCO_3_ ± 1 or 10 µg/ml gentamicin (Gen) or ± 1 or 12.5 µg/mL tobramycin (Tob). (**C**) *S. aureus* Δ*menD* was grown at 37°C for 5 h prior to serial dilution and plating on TSA +14 mM glucose ± 25 mM NaHCO_3_ ± 7.5 µg/mL gentamicin, followed by growth at 37°C until CFUs could be enumerated. Data are presented as mean ± SD of six biological replicates. One-way ANOVA was used to determine statistical significance where ns, *P* > 0.05; ***P < 0.001; ****P < 0.0001. (**D**) *S. aureus* Δ*menD* was grown at 37°C for 5 h prior to serial dilution and plating on TSA +14 mM glucose ± 25 mM NaHCO_3_ ± 15 µg/mL tobramycin, followed by growth at 37°C until CFUs could be enumerated. Data are presented as mean ± SD of four biological replicates. One-way ANOVA was used to determine statistical significance where ns, *P* > 0.05; ****P < 0.0001.

We then performed an aminoglycoside susceptibility assay using a spot dilution test. 25 mM bicarbonate alone does not affect the CFUs of Δ*menD* on solid medium (Fig. 6; Fig. S6 and S7). JE2 is severely compromised for growth on plates containing 1 µg/mL of the aminoglycosides gentamicin or tobramycin (Fig. 6A; Fig. S6 and S7). Consistent with previous work from our group and others, Δ*menD*, *pbgS::Tn*, and *qoxB::Tn* Δ*cydB* are tolerant to aminoglycosides compared to JE2 due to a collapsed PMF (Fig. S6 and S7) (44, 45), and Δ*menD* shows the highest levels of tolerance to gentamicin and tobramycin (Fig. 6B; Fig, S6 and S7). When Δ*menD* is plated to media containing 10 µg/mL gentamicin and 25 mM bicarbonate, gentamicin tolerance is decreased by greater than 1,000-fold (Fig. 6B). Bicarbonate also decreases tobramycin tolerance in Δ*menD* (Fig. 6B). Gentamicin and tobramycin tolerance in *pbgS::Tn* and *qoxB::Tn* Δ*cydB* decreases on plates containing 25 mM bicarbonate as well (Fig. S6 and S7). Dilution plating confirmed that the combinatory treatment of bicarbonate and aminoglycoside increases killing of Δ*menD* compared to aminoglycoside alone by 1,000-fold for gentamicin (Fig. 6C), and 20-fold for tobramycin (Fig. 6D). We observed that 25 mM bicarbonate in liquid medium significantly decreases CFUs of Δ*menD* (Fig. 1B and D), but 25 mM bicarbonate in solid medium does not decrease the CFUs of Δ*menD* (Fig. 6C and D), indicating that the Δ*menD* is more sensitive to bicarbonate in liquid medium. These results demonstrate that bicarbonate potentiates aminoglycoside susceptibility in SCVs.

**Fig 7:**
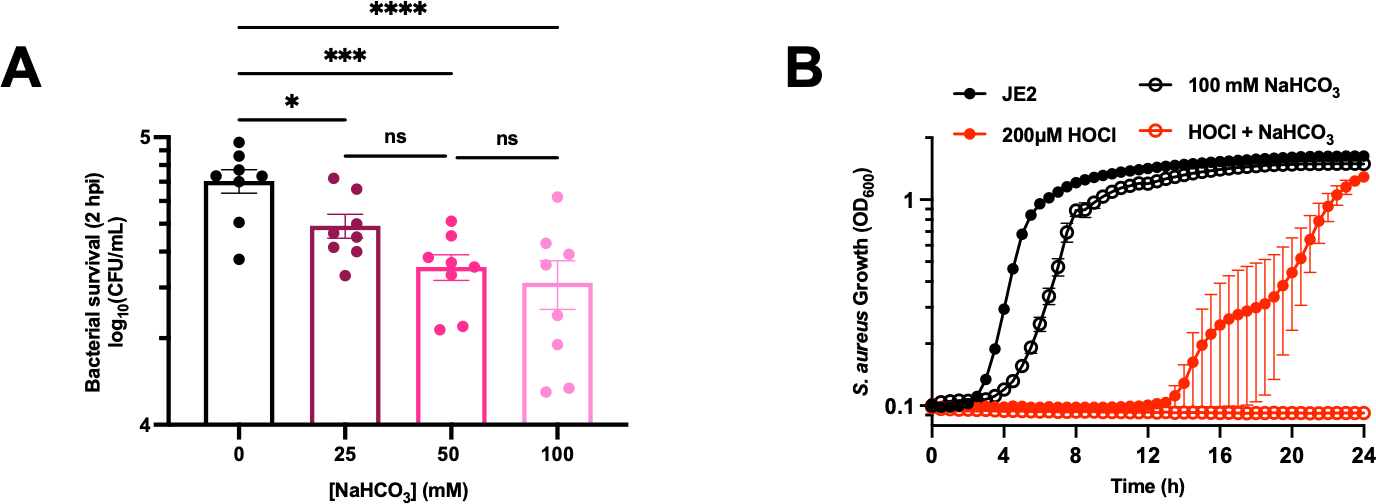
Bicarbonate sensitizes *S. aureus* JE2 to murine neutrophil killing. (**A**). Neutrophils isolated from wild-type C57BL/6J mice were infected with *S. aureus* JE2 grown in various concentrations of bicarbonate (MOI = 10) and incubated for 2 h. After incubation, the neutrophils were lysed and the bacteria were diluted and plated for CFU enumeration. Data are presented as mean ± SD of three independent experiments with three biological replicates in each experiment (*n* = 9). One-way ANOVA was used to determine statistical significance where ns, *P* < 0.05; ***P < 0.001; ****P < 0.0001. (**B**) JE2 was treated ± 200 µM HOCl in PBS for 40 min, then added to tryptic soy broth (TSB) + HEPES ± 100 mM NaHCO_3_. Growth was measured by monitoring the OD_600_ every 30 min for 24 h. Data are presented as mean ± SEM of six biological replicates.

### Bicarbonate sensitizes *S. aureus* JE2 to hypochlorous acid (HOCl), enhancing neutrophil killing

Bicarbonate is an abundant component in human blood, and we therefore tested the effects of bicarbonate on host killing of *S. aureus* by pretreating JE2 with increasing concentrations of bicarbonate prior to infecting murine neutrophils. During the infection, *S. aureus* and neutrophils were further exposed to 24 mM bicarbonate from the cell culture medium. Bicarbonate sensitizes *S. aureus* to neutrophil killing dose-dependently (Fig. 7A). Even physiological concentrations of bicarbonate (25 mM) sensitize *S. aureus* to neutrophil killing (Fig. 7A). A major route of *S. aureus* killing by neutrophils is through the enzymatic activity of myeloperoxidase that produces HOCl, a potent oxidant (53, 54). Pretreatment of JE2 with HOCl enhances *S. aureus* sensitivity to bicarbonate (Fig. 7B). These data demonstrate that bicarbonate makes *S. aureus* more susceptible to neutrophil killing via potential cooperativity with the antimicrobial activity of HOCl. They also suggest that bicarbonate in the blood plays an important role in aiding the immune system in clearing *S. aureus* infections from the host.

## DISCUSSION

The highest concentration of bicarbonate in the human body, 150 mM, is secreted by the pancreas (29). Pancreatic bicarbonate neutralizes gastric acid in the stomach, providing an optimal pH for digestive enzymes (30). Systemically, bicarbonate is the major buffer of body fluids, protecting against toxic pH fluctuations (30). Clinical trials demonstrated that bicarbonate is an effective treatment for metabolic acidosis, sepsis, cystic fibrosis, and cardiac arrhythmias (29, 33, 55, 56). The dental and food preparation industries have used bicarbonate as a microbial control agent for a century without completely understanding its mechanism of action (31). The impact of bicarbonate on *S. aureus* SCVs has also not been described previously.

Bicarbonate also plays an important role in providing DIC for many metabolic processes in bacteria, including *S. aureus* (39). Deletion of the MpsAB transporter causes a severe growth defect in *S. aureus* (37) that is rescued by bicarbonate supplementation (37). MpsAB is the only reported DIC transporter, which provides bicarbonate for anaplerotic metabolism (38), and a carbonic anhydrase has not been reported in *S. aureus* (38). During bicarbonate anaplerotic metabolism, Pyc combines bicarbonate with pyruvate to generate oxaloacetate (49), which fuels the TCA cycle, amino acids biosynthesis, and purine biosynthesis (57). We discovered that *S. aureus* SCVs are highly sensitive to bicarbonate stress compared to the wild-type parental JE2 strain and hypothesized that the bicarbonate anaplerotic reaction has a role in bicarbonate detoxification. We quantified ^13^C-oxaloacetate levels in strains treated with NaH^13^CO_3_ and discovered that Δ*menD* has significantly lower levels of ^13^C-oxaloacetate compared to JE2, indicating defective anaplerotic metabolism in SCVs. To verify that defective bicarbonate anaplerotic metabolism is indeed involved in the bicarbonate toxicity of SCVs, we measured the ^13^C-oxaloacetate levels in a Pyc-deficient strain. Inactivating *pyc* increases bicarbonate sensitivity compared to wild type. The Pyc-deficient strain grown with NaH^13^CO_3_ also has significantly lower levels of ^13^C-oxaloacetate compared to the wild type grown with NaH^13^CO_3_. These data collectively demonstrate that defective bicarbonate anaplerotic metabolism sensitizes *S. aureus* SCVs to bicarbonate, indicating that targeting this metabolic pathway could help resolve persistent *S. aureus* infections in humans.

To understand the mechanism of defective anaplerotic metabolism in SCVs, we measured Pyc expression in wild type and SCVs. Δ*menD* grown with bicarbonate has increased levels of Pyc compared to wild type, indicating that bicarbonate does not suppress Pyc expression in SCVs, but induces it, hinting at its role in bicarbonate detoxification. We then measured pyruvate levels, finding that Δ*menD* has lower levels of pyruvate compared to wild type. Supplementation of the growth medium with exogenous pyruvate protects against bicarbonate toxicity in SCVs. Further confirming the role of pyruvate depletion in bicarbonate sensitivity of SCVs, the combined treatment of pyruvate and NaH^13^CO_3_ increases ^13^C-oxaloacetate levels higher than what is observed in NaH^13^CO_3_-treated JE2. However, we observed higher levels of ^13^C-oxaloacetate in SCVs with pyruvate and NaH^13^CO_3_ at 2.5 h, but not at 5 h. It is reported that transamination of oxaloacetate can produce aspartate, a key precursor for amino acid and purine biosynthesis (51, 57, 58). Since SCVs do not run the TCA cycle nor oxidative phosphorylation (46), it is possible that when pyruvate is replete, SCVs use bicarbonate and pyruvate to make oxaloacetate, which is rapidly converted to aspartate and other downstream metabolites of amino acid and purine biosynthesis (51, 57, 58). These findings indicate that low levels of cellular pyruvate are responsible for the defects in bicarbonate anaplerotic metabolism observed in SCVs.

SCVs grown with bicarbonate experience a greater increase in cytoplasmic pH compared to wild type. These findings indicate wild type maintains a steady-state cytoplasmic pH upon bicarbonate treatment, while the cytoplasmic pH of SCVs continues to rise without reaching a steady state. Alkalinization of the cytoplasmic pH is toxic to bacterial cells (59). Based on this study, our findings suggest that SCVs are unable to compensate for the bicarbonate alkaline toxicity in the cytoplasm because of a defective bicarbonate anaplerotic metabolism caused by low levels of pyruvate. As a result, bicarbonate causes alkaline toxicity in the cytoplasm and inhibits the growth of SCVs. In anaerobic conditions, the wild type neither runs the TCA cycle nor performs oxidative phosphorylation, relying solely on glycolytic metabolism to produce energy and phenotypically turns into an SCV (47). Bicarbonate significantly inhibits the growth of wild type in anaerobic conditions, suggesting that bicarbonate anaplerotic metabolism is also defective in anaerobically grown *S. aureus*. Consistent with our findings, pyruvate is essential for *S. aureus* to survive in the anaerobic environment of host bone marrow (57). This study, combined with our data, indicates that bicarbonate may be an important host factor in preventing osteomyelitis infections due to alterations to bicarbonate anaplerotic metabolism anaerobically.

 SCVs are highly tolerant to aminoglycoside antibiotics, making them particularly difficult to treat (45). SCVs maintain a basal cytoplasmic pH of 7.38 compared to a basal cytoplasmic pH of 7.9 in wild type when both are exposed to an extracellular pH of 7.30. These results indicate that SCVs have a nearly similar pH inside and outside of the cell, and thus a diminished pH gradient across the membrane. Aminoglycosides enter bacterial cells dependent upon the presence of a pH gradient (31), and our data demonstrate a diminished pH gradient as driving SCV tolerance of aminoglycosides. Bicarbonate alkalinizes the cytoplasm of SCVs, restoring the pH gradient across the cellular membrane and aminoglycoside susceptibility in SCVs. Consistent with our findings, another study reported that alkalinization of growth media with arginine increases aminoglycoside susceptibility of *S. aureus* (52). Alkalinization of the cytoplasm was also reported as a strategy to increase norfloxacin activity against *E. coli* (60). In total, bicarbonate is an effective potentiator of antibiotic activity, and our data demonstrate that alkalinizing the cytoplasm of SCVs is a potential therapeutic strategy to increase antibiotic susceptibility in persistent *S. aureus* strains.

We also tested the role of bicarbonate at the host-pathogen interface through neutrophil killing of *S. aureus*. JE2 treated with bicarbonate is more sensitive to neutrophil killing compared to untreated JE2. HOCl is a major antimicrobial component of neutrophils (53, 54), and cooperatively kills *S. aureus* with bicarbonate. These data demonstrate that bicarbonate enhances neutrophil-mediated killing of *S. aureus* by enhancing the effects of HOCl. Consistent with these findings, another group showed that bicarbonate enhances the antimicrobial activity of several components, such as LL-37, indolicidin, α-defensin, bile salts, hyaluronic acid, bactenesin, protegrin, and lysozyme, of the host innate immunity (31). Moreover, this finding suggests that the presence of bicarbonate in host blood may help the immune system to clear *S. aureus*.

**Fig 8:**
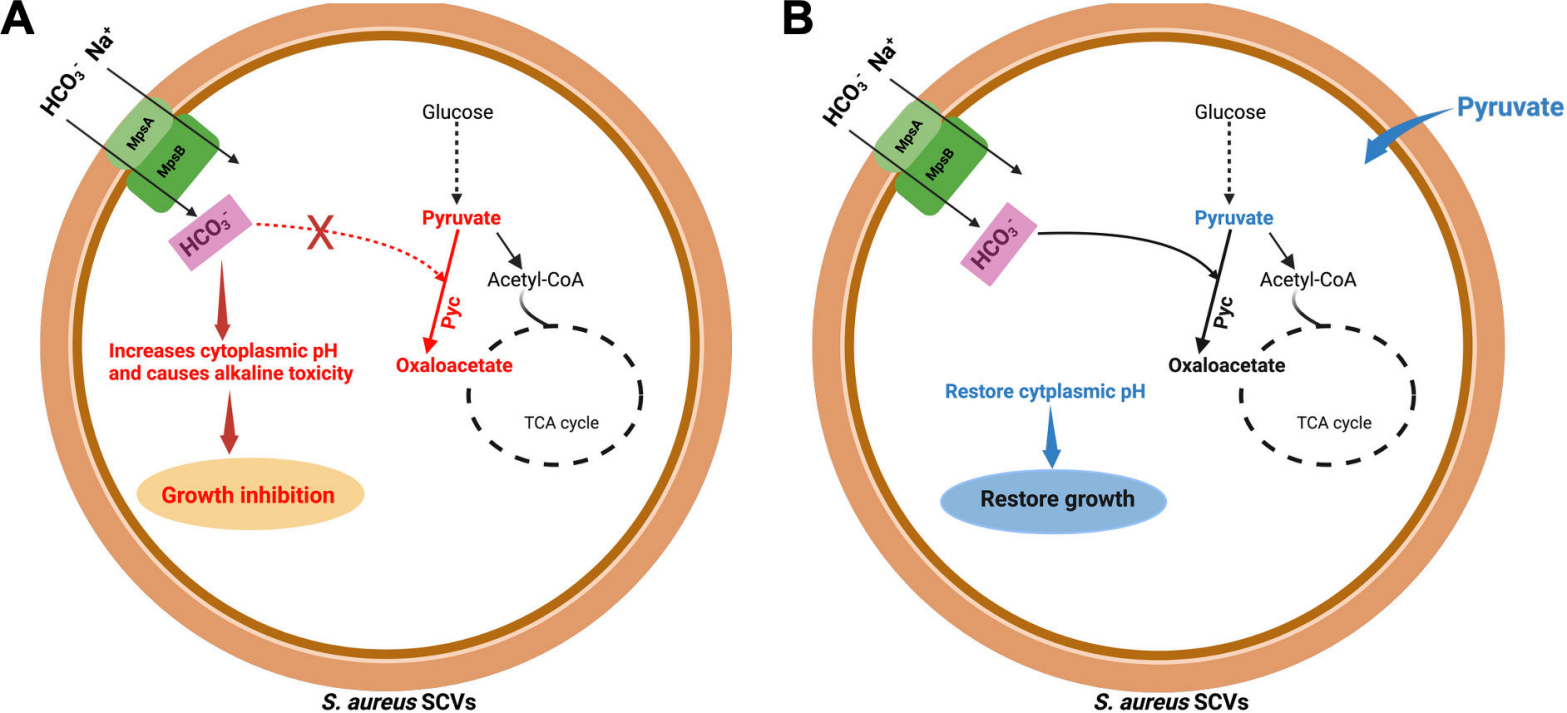
Defects in anaplerotic metabolism sensitize *S. aureus* SCVs to bicarbonate. (**A**) SCVs have defective bicarbonate anaplerotic metabolism due to low levels of pyruvate. As a result, bicarbonate increases the cytoplasmic pH, generating alkaline toxicity and inhibiting the growth of SCVs. (**B**) Exogenous pyruvate provides ample substrate, facilitating bicarbonate anaplerotic metabolism in SCVs, restoring the cytoplasmic pH, and protecting the SCVs from bicarbonate toxicity.

Here, we report that SCVs have defective anaplerotic metabolism due to low levels of pyruvate, resulting in cytoplasmic alkalinization and cellular toxicity (Fig. 8A). Exogenous pyruvate restores bicarbonate anaplerotic metabolism, lowering the cytoplasmic pH and preventing bicarbonate toxicity in SCVs (Fig. 8B). Previously, a clinical study reported that serum bicarbonate and pH are decreased in sepsis patients, and that low levels of patient serum bicarbonate are associated with elevated hospital mortality of sepsis patients (61). Based on this clinical study, our findings suggest that *S. aureus* may acquire bicarbonate from the host during infection to support the synthesis of oxaloacetate, which will feed into the TCA cycle or drive amino acid, purine, and cell wall biosynthesis (51, 57, 58), thereby promoting the spread of infection in the host. The depletion of bicarbonate and other nutrients at the infection site may create a protected niche where *S. aureus* can shift its metabolism to form SCVs, leading to chronic infection in the host. Our study defines that SCVs are sensitive to bicarbonate due to a defect in anaplerotic metabolism caused by low levels of cellular pyruvate. Bicarbonate and pyruvate levels vary across host tissues (29, 62), indicating that the role of bicarbonate anaplerotic metabolism to cause *S. aureus* infection may also vary based on the site of infection (63). For instance, previous studies report that the kidneys of healthy individuals contain high levels of pyruvate (62), and that kidney is the primary site for abscess formation during sepsis (64). It was also reported that pyruvate enhances the virulence of *S. aureus* (65). These findings suggest that in host niches where pyruvate is abundant, such as the kidneys, *S. aureus* effectively uses bicarbonate to support bicarbonate anaplerotic metabolism and neutralize the bicarbonate toxicity. Alternatively, our study, along with others, indicates that inhibition of pyruvate acquisition from the host and pyc-driven bicarbonate anaplerotic metabolism could be a promising therapeutic strategy to control *S. aureus* infection in the host (62, 64, 65). Indeed, this concept is further supported by a previous study showing that inhibition of pyruvate synthesis and Pyc-driven bicarbonate anaplerotic metabolism significantly decreases *S. aureus* infection in bone tissue (57). Overall, the results of our study define the role of bicarbonate anaplerotic metabolism in protecting *S. aureus* from bicarbonate toxicity and highlight a possible therapeutic target for the treatment of persistent infection caused by *S. aureus* SCVs.

## MATERIALS AND METHODS

### Growth medium, plasmid, bacterial strains, and chemicals

All bacterial strains and plasmids used in this study are listed in Table 1. All plasticware and reagents were purchased from VWR (Radnor, PA), unless otherwise noted. Difco tryptic soy broth (TSB) and Difco tryptic soy agar (TSA) growth media were purchased from VWR. Growth curve and CFU experiments were performed in 96-well plates purchased from Falcon Brand, Corning Incorporated (Kennebunk, ME). Antibiotics were purchased from either VWR or Sigma-Aldrich and used in the following concentrations: carbenicillin (Cb) 50 µg/mL (for selection of *E. coli* DH5α), chloramphenicol (Chl) 10 µg/mL, and erythromycin (Erm) 10 µg/mL. Gentamicin (Gen) was purchased from Quality Biologicals (Gaithersburg, MD). Tobramycin (Tob), 1-ethyl-3-(3-dimethylaminopropyl)carbodiimide (EDC), MES buffer, CHES buffer, and IGEPAL CA-630 were purchased from ThermoFisher Scientific (Waltham, MA). Anhydrotetracycline (anTet) was purchased from AdipoGen Life Sciences (San Diego, CA). Hemin chloride was purchased from Calbiochem (San Diego, CA). Sodium bicarbonate (NaHCO_3_), tris buffer, HPLC-grade ethanol, HPLC-grade ethyl acetate, and phenylmethyl sulfonyl fluoride (PMSF) were purchased from VWR. NaH^13^CO_3_ was purchased from Cambridge Isotope Laboratories, Inc. (Tewksbury, MA). Sodium pyruvate was purchased from BeanTown Chemicals (Hudson, NH). Pyridine, LC-MS-grade water, LC-MS-grade acetonitrile, LC-MS-grade formic acid, and HPLC-grade methanol were purchased from Fisher Chemicals (Pittsburgh, PA). O-benzyl hydroxyl amine (OBHA) was purchased from Tokyo Chemical Industry (Portland, OR). Carbonyl cyanide *m-*chlorophenyl hydrazone (CCCP) was purchased from Cayman Chemicals (Ann Arbor, MI). All oligonucleotide primers used in this study were purchased from IDT (Coralville, IA) and are listed in Table S1. GeneJET genomic DNA purification kit, GeneJET plasmid miniprep kit, and GeneJET PCR purification kit were purchased from ThermoFisher Scientific.

**TABLE 1:** Bacterial strains and plasmids

Strain or plasmid	Description	Source
*S. aureus* strains		
JE2	Wild-type, USA300 community-acquired methicillin-resistant *S. aureus* (CA-MRSA) isolate	(41)
RN4220	Wild-type, restriction enzyme-deficient cloning intermediate strain	(66)
Δ*menD*	USA300 Δ*menD*, in-frame *menD* (*SAUSA300_0946*) deletion with allele exchange	This study
*ΔmenD*	JE2 Δ*menD*, in-frame *menD* (*SAUSA300_0946*) deletion with allele exchange	This study
*qoxB::Tn*	JE2 *qoxB::Tn* (NE732; *SAUSA300_0962::Tn*)	(41)
*qoxB::Tn* Δ*cydB*	JE2 *qoxB::Tn* Δ*cydB*, in-frame *cydB* (*SAUSA300_0987*) deletion with allele exchange in *qoxB::Tn* background	This study
*pbgS::Tn*	JE2 *pbgS::Tn* (NE1845; *SAUSA300_1615::Tn*)	(41)
*pyc::Tn*	JE2 *pyc::Tn* (NE754; *SAUSA300_1014::Tn*)	(41)
*E. coli* strain		
DH5a	Plasmid cloning intermediate strain	(67)
Plasmid		
pKOR1	Allele exchange vector for gene deletion, Chl^R^	(15, 68)
pKOR1.Δ*menD*	Allele exchange vector for *menD* deletion, Chl^R^	This study
pKOR1.Δ*cydB*	Allele exchange vector for *cydB* deletion, Chl^R^	This study
pOS1.P*_lgt_*	Expression vector with *lgt* constitutive promoter, Chl^R^	(15, 69)
pOS1.P*_lgt_.menD*	pOS1.Igt vector constitutively expressing *menD*, Chl^R^	This study
pOS1.P*lgt.pyc*	pOS1.Igt vector constitutively expressing *pyc*, Chl^R^	This study
pOS1.P*_lgt_.cydB*	pOS1.Igt vector constitutively expressing *cydB*, Chl^R^	This study

### Deletion of *menD* and *cydB* from *S. aureus* strains

The *menD* gene in *S. aureus* JE2 background was deleted using the previously described pKOR1 allelic exchange method (68) with some modifications. NEbuilder tool (New England Biolab) was used to design the primers to amplify DNA for Gibson Assembly of the pKOR1 plasmid. The 1 kb upstream region of *menD* was PCR amplified using the primer pair, menD-up_Fw and menD-up_Rv, and the 1 kb downstream region of *menD* was PCR amplified using the primer pair, menD-dw_Fw and menD-dw_Rv, from *S. aureus* JE2 genomic DNA. The pKOR1 plasmid was linearized by PCR using the primer pair WNB00047 and WNB00048. PCR-amplified pKOR1, upstream, and downstream regions of *menD* were assembled to produce the pKOR1.Δ*menD* construct using Gibson Assembly HiFi Master Mix (Life Technologies Corporation). The pKOR1.Δ*menD* construct was introduced into chemically competent *E. coli* DH5α using a previously described heat shock method (66). The pKOR1.Δ*menD* construct was isolated from transformed *E. coli* DH5α and confirmed by PCR using the primer pair WNB00058 and WNB00059. The construct was then introduced into *S. aureus* RN4220 before being introduced into *S. aureus* JE2 via electroporation (66). The deletion of *menD* in *S. aureus* JE2 was confirmed by PCR using primer menD-KO_Fw and menD-KO_Rv. The *menD* gene was also deleted in *S. aureus* USA300 background using the same methods and primers used for the deletion of *menD* from *S. aureus* JE2 background. The deletion of *cydB* from *S. aureus qoxB::Tn* (JE2 background) was also performed using the above protocol, and the primers used for deletion of *cydB* from the JE2 *qoxB::Tn* background are listed in Table S1. Δ*menD* in the USA300 background, Δ*menD* in the JE2 background, and *qoxB::Tn* Δ*cydB* in the JE2 background strains were whole genome sequenced at SeqCoast Genomics (Newington, NH) to confirm the deletion and to ensure that off-target mutations did not occur.

### Construction of pOS1.P*_Igt_*.*menD*, pOS1.P*_Igt_*.*cydB,* and pOS1.P*_Igt_*.*pyc*

For complementation of *S. aureus* Δ*menD* phenotypes, *menD* was cloned into an expression vector pOS1 under lipoprotein diacylglyceryl transferase (*Igt*) constitutive promoter (69). The pOS1.P*_Igt_* plasmid was linearized by PCR using the primer pair WNB00137 and WNB00138. The *menD* gene was PCR amplified from genomic DNA of *S. aureus* JE2 using the primer pair menD_Fw and menD_Rv. The PCR-amplified pOS.P*_Igt_* and *menD* were assembled to produce the pOS1.P*_Igt_.menD* plasmid using Gibson Assembly HiFi Master Mix. The pOS1.P*_Igt_.menD* construct was introduced into chemically competent *E. coli* DH5α using a heat shock method (66). The construct was isolated from transformed *E. coli* DH5α and confirmed by PCR using the primer pair WNB00080 and WNB00081. The construct was then introduced into *S. aureus* RN4220 via electroporation and moved into *S. aureus* Δ*menD* via electroporation, which is described below. The pOS1.P*_Igt_.cydB* construct was also prepared using the above protocol and transferred into *S. aureus qoxB::Tn* Δ*cydB* by Φ85 bacteriophage transduction, which is described below. The primers used for *cydB* cloning are listed in Table S1.

For complementation of JE2 *pyc::Tn* phenotypes, *pyc* was cloned into pOS.P*_Igt_* plasmid vector under the constitutive control of the *Igt* promoter. The alternative TTG start codon of *pyc* was changed to ATG and amplified by PCR from genomic DNA of *S. aureus* JE2 using the primer pair pyc_Fw and pyc_Rv. The PCR-amplified pOS.P*_Igt_* and *pyc* were assembled to produce pOS1.P*_Igt_.pyc* construct using Gibson Assembly HiFi Master Mix. The pOS1.P*_Igt_.pyc* construct was introduced into chemically competent *E. coli* DH5α using a heat shock method (66). The construct was isolated from transformed *E. coli* DH5α and confirmed by PCR using the primer pair WNB00080 and WNB00081. The construct was then introduced into *S. aureus* RN4220 via electroporation and moved into *S. aureus pyc::Tn* via electroporation, which is also described below.

### Preparation of *S. aureus* JE2- and Δ*menD*-competent cells for electroporation

JE2- and Δ*menD*-competent cells for electroporation were prepared using the previously described method (70) with some modifications. A single colony of *S. aureus* JE2 was inoculated into 5 mL of TSB, while a single colony of *S. aureus* Δ*menD* was inoculated into 5 mL of TSB containing 5 µM menadione and grown at 37°C overnight in a shaking incubator (Innova 42, New Brunswick, Eppendorf, Hamburg, Germany). The overnight culture of JE2 was freshly diluted 100-fold into 30 mL of TSB, while the overnight culture of Δ*menD* was diluted 100-fold into 30 mL of TSB containing 5 µM menadione. The cultures were grown for approximately 3 h at 37°C in a shaking incubator until an optical density (OD_600_) of 0.6 was reached. The cultures were then pelleted by centrifugation (Mega Star 4.0R, VWR, Radnor, PA) at 4,000 rpm at 4°C. The supernatant was discarded, and the cells were washed two times with 10% glycerol. Later, the cells were resuspended in 3 mL of 10% glycerol, aliquoted, and stored in a −80°C freezer.

### Transformation of pOS1.P*_Igt_*.*menD* into *S. aureus* strains

The pOS1.P*_Igt_* and pOS1.P*_Igt_.menD* purified from *S. aureus* RN4220 was introduced into *S. aureus* JE2 and Δ*menD* via electroporation. The 100 µL of competent cells of *S. aureus* JE2 and Δ*menD* was electroporated with approximately 200 ng of either pOS1.P*_Igt_* control plasmid or pOS1.P*_Igt_.menD* construct plasmid. After pulsing, the transformed *S. aureus* JE2 and Δ*menD* cells were immediately supplemented with 1 mL of TSB containing 0 and 5 µM menadione, respectively. The cells were transferred into 1.5 mL microcentrifuge tubes and incubated for 1 h at 37°C in a shaking incubator. The cells were centrifuged, resuspended in 150 µL TSB, and spread onto a TSA plate containing 10 µg/mL chloramphenicol. The transformation of pOS1.P*_Igt_* and pOS1.P*_Igt_.menD* into *S. aureus* JE2 and Δ*menD* was confirmed by PCR using primers WNB00080 and WNB00081.

### Transformation of pOS1.P*_Igt_*.*pyc* into *S. aureus* strains

The electroporation method was also used for the transformation of *S. aureus* JE2 and *pyc::Tn* with pOS1.P*_Igt_* and pOS1.P*_Igt_.pyc* plasmid vector. 100 µL of competent cells of *S. aureus* JE2 and *pyc::Tn* was electroporated with approximately 200 ng of either pOS1.P*_Igt_* control plasmid or pOS1.P*_Igt_.pyc* construct plasmid. After electroporation, the transformed *S. aureus* JE2 and *pyc::Tn* cells were immediately supplemented with 1 mL of TSB. The rest of the transformation process is described in the above section. The transformation of pOS1.P*_Igt_* and pOS1.P*_Igt_.pyc* into *S. aureus* JE2 and *pyc::Tn* was confirmed by PCR using primers WNB00080 and WNB00081.

### Transduction of pOS1.P*_Igt_*.*cydB* into *S. aureus* strains

The pOS1.P*_Igt_* and pOS1.P*_Igt_.cydB* plasmids purified from *S. aureus* RN4220 were introduced into *S. aureus* JE2 via electroporation. Φ85 bacteriophage transduction was used to transfer pOS1.P*_Igt_* and pOS1.P*_Igt_.cydB* plasmids from RN4220 into *S. aureus qoxB::Tn* Δ*cydB. S. aureus* RN4220 with either pOS1.P*_lgt_* or pOS1.P*_Igt_.cydB* was inoculated into aeration culture tubes containing 5 mL of TSB supplemented with 10 µg/mL chloramphenicol and grown overnight at 37°C in a shaking incubator. 250 µL of Φ85 bacteriophage lysate (propagated in *S. aureus* JE2) and 250 mL overnight culture of donor RN4220 with either pOS1.P*_lgt_* or pOS1.P*_Igt_.cydB* were added to 15 mL screw cap tubes containing 5 mL of TSB and 5 mL of phage buffer (1 mM MgSO_4_, 4 mM CaCl_2_, 50 mM Tris-HCl, 100 mM NaCl, and 0.1% gelatin). The tubes were incubated at room temperature overnight without shaking. The Φ85 supernatant was filter sterilized and stored at 4°C. 1,000, 100, 10, 0 µL of Φ85 lysates were added into 15 mL screw cap tubes. 1 mL of *qoxB::Tn* Δ*cydB* overnight was added to each tube. The tubes were incubated at 37°C for 20 min. After incubation, 2 mL of 34 mM sodium citrate was added to each tube and mixed well. The tubes were centrifuged, the supernatants were discarded, and bacterial cells were resuspended in 2 mL of TSB containing 17 mM sodium citrate. The bacterial cells were incubated at 37°C for 1 h. The bacterial cells were then centrifuged, the supernatants were discarded, and the cells were resuspended in 1 mL of TSB containing 17 mM sodium citrate. 100 µL and 900 µL of each bacterial sample were spread onto TSA containing 17 mM sodium citrate and 10 µg/mL chloramphenicol, and the plates were incubated at 37°C for 72 h. Single colonies of *qoxB::Tn* Δ*cydB* transduced with either pOS1.P*_lgt_* or pOS1.P*_Igt_.cydB* were streaked two times onto TSA containing 17 mM sodium citrate and 10 µg/mL chloramphenicol. The transformation of pOS1.P*_Igt_* and pOS1.P*_Igt_.cydB* into *S. aureus* JE2 and *qoxB::Tn* Δ*cydB* was confirmed by PCR using primers WNB00080 and WNB00081.

### Preparation of *S. aureus* bacterial culture

For all experiments, unless otherwise noted, *S. aureus* strains were grown in TSB or on TSA. *S. aureus* JE2 and SCVs (Δ*menD*, *pbgS::Tn*, and *qoxB::Tn* Δ*cydB*) were streaked to TSA from −80°C freezer stocks and grown at 37°C for 24 h and 48 h, respectively. Single colonies of *S. aureus* JE2 and SCV strains were inoculated into aeration culture tubes containing 5 mL of TSB and grown at 37°C for 24 h in a shaking incubator at 180 rpm.

### Preparation of TSB + HEPES medium

The addition of sodium bicarbonate to TSB or TSA increases the pH. Therefore, TSB and TSA were buffered with 100 mM HEPES buffer (pH 7.30). The media was autoclaved at 121°C at 15 psi for 15 min.

### *S. aureus* growth analysis with or without sodium bicarbonate

Bicarbonate growth was measured in 96-well plates with six biological replicates per condition tested. *S. aureus* JE2 and SCV (Δ*menD*, *pbgS::Tn*, and *qoxB::Tn* Δ*cydB*) strains were diluted 1,000- and 100-fold, respectively, into TSB + HEPES media supplemented with 0 or 50 mM sodium bicarbonate in a 96-well plate. The growth measurement was performed in an EPOCH 2 (BioTek, Winooski, VT) plate reader, taking OD_600_ readings every 30 min for 24 h at 37°C with linear shaking at 567 cpm (3 mm) (15).

### *S. aureus* CFU analysis with or without bicarbonate

*S. aureus* JE2 and SCV (Δ*menD*, *pbgS::Tn*, and *qoxB::Tn* Δ*cydB*) strains were diluted 1,000- and 100-fold into TSB + HEPES media supplemented with 0 or 50 mM sodium bicarbonate in a 96-well plate. The plate was analyzed in an EPOCH 2 plate reader, taking OD_600_ readings every 30 min for 17 h at 37°C with linear shaking at 567 cpm (3 mm). 10 µL of each well was removed after 0 and 17 h of incubation and serially diluted in PBS (phosphate buffer saline, pH 7.30) in 10-fold intervals from 10^0^ to 10^−6^. 10 µL of each dilution was spotted onto TSA and incubated overnight at 37°C, followed by CFU enumeration.

### *S. aureus* bicarbonate sensitivity in anaerobic conditions

*S. aureus* JE2 was streaked onto TSA from the −80°C freezer stock and grown at 37°C for 48 h in an anaerobic chamber (Coy, Ann Arbor, MI). Single colonies of *S. aureus* JE2 were inoculated into 5 mL of TSB and grown statically at 37°C overnight in an anaerobic chamber. JE2 was diluted 1000-fold in TSB + HEPES media containing 0 or 100 mM sodium bicarbonate and 0 or 100 mM sodium nitrate in a 96-well plate. The plate was incubated statically at 37°C for 17 h in an anaerobic chamber. 10 µL of bacteria was removed from each well and serially diluted in 10-fold intervals from 10^0^ to 10^−6^ in PBS. 10 µL of each dilution was spotted onto TSA and incubated at 37°C for 24 h in the anaerobic chamber, followed by CFU enumeration.

### *S. aureus* growth with or without sodium bicarbonate and sodium pyruvate

*S. aureus* JE2 and SCV (Δ*menD*, *pbgS::Tn*, and *qoxB::Tn* Δ*cydB*) strains were diluted 1,000- and 100-fold, respectively, into 100 µL of TSB + HEPES media containing 0 or 50 mM sodium bicarbonate and 0 or 4.5 mM sodium pyruvate in a 96-well plate. The growth analysis was performed in the EPOCH 2 plate reader as described above (15).

### ^13^C- and ^12^C-oxaloacetate extraction and derivatization

Single colonies of *S. aureus* JE2 and Δ*menD* were inoculated into aeration culture tubes containing 5 mL of TSB supplemented with 0 or 9 mM sodium pyruvate and grown at 37°C for 24 h in a shaking incubator. Overnight cultures of JE2 and Δ*menD* were diluted 100- and 10-fold in aeration culture tubes containing 1 mL of TSB + HEPES media supplemented with 0 or 50 mM NaH^13^CO_3_ and 0 or 18 mM sodium pyruvate. The bacterial cultures were grown for either 2.5 or 5 h at 37°C in a shaking incubator. The cells were pelleted by centrifugation, then suspended in 190 µL of 10 mM MgSO_4_ in PBS and 10 µL of 2 mg/mL lysostaphin. The samples were incubated for 10 min at 37°C. 400 µL of ice-cold methanol was added to each tube, then sonicated for 10 seconds with a probe sonicator (Model FB120, Fisher Scientific, Hampton, NH) on a setting pulse 10 sec and amplitude 50%. Insoluble cellular debris was pelleted by centrifugation for 5 min at 20,817 rcf and 4°C, and total protein was quantified from the supernatants by BCA protein assay kit (Millipore Sigma, 71285-3). The oxaloacetate in the supernatant was derivatized by adding 1 mL of pyridine reaction buffer (21.6 mL of 6 N HCl, 17.2 mL of pyridine, and 161.2 mL of water, pH 5.0), 50 µL of 1 M EDC, and 50 µL of 1 M O-BHA to each sample. The tubes were incubated at room temperature for 1 h. The derivatized analytes were extracted thrice with 3 mL of ethyl acetate, and the combined extracts were dried in a nitrogen gas evaporator (Organomation, Los Angeles, CA). The extracted samples were dissolved in 100 µL of methanol and 50 µL of water for LC-MS/MS analysis.

### Pyruvate extraction and derivatization

JE2 and Δ*menD* overnights were diluted 100- or 10-fold, respectively, into aeration culture tubes containing 1 mL of TSB + HEPES and grown for 5 h at 37°C in a shaking incubator. The cells were then transferred to a 1.5 mL tube and centrifuged for 2 min at 20817 rcf at 4°C. The cell lysis, derivatization, and extraction were performed using the same methods described above for oxaloacetate.

### LC-MS/MS analysis of oxaloacetate and pyruvate

The samples were analyzed at the LSU School of Veterinary Medicine Mass Spectrometry Resource Center on a Shimadzu 8060 NX triple quadrupole mass spectrometer interfaced with a Shimadzu Nexera XS 40 series UHPLC and Shimadzu CTO-40S column oven. 10 µL of each derivatized sample, blank, or standard mixture was analyzed in positive ionization mode. Chromatographic separation was achieved using a Phenomenex Kinetex C_8_ (150 mm length, 2.1 mm internal diameter, 2.6 µm particle size, 100 Å pore size) column with a Phenomenex Security Guard C_8_ (2.1 mm internal diameter) guard column. Mobile phase A was 0.1% formic acid in water, and mobile phase B was 0.1% formic acid in acetonitrile. The column oven was set to 25°C, and the flow rate was set to 0.4 mL/min for the entire analysis. The starting condition was 10% B, which was held for 2 min. Mobile phase B was then ramped to 50% over the next 23 min. The column was washed at 98% B for 10 min, then equilibrated to 10% B for 5 min. All instrument voltages were determined and optimized empirically prior to the analysis. The following *m/z* transitions were monitored for the loss of O-BHA: 448.2–91.0 for ^12^C-oxaloacetate, 449.2–91.0 for ^13^C-oxaloacetate, and 299.1–91.0 for pyruvate. A calibration line of each derivatized standard was used to quantify each sample. ^12^C-oxaloacetate and ^13^C-oxaloacetate were compared to determine isotopic enrichment for each sample. Pyruvate levels were normalized to total protein for differences in starting material.

### Proteomic analysis

Overnight cultures of *S. aureus* USA300 and Δ*menD* strains were diluted 16-fold into aeration culture tubes containing 5 mL of TSB + HEPES media supplemented with 0 or 150 mM sodium bicarbonate. The culture tubes were incubated at 37°C for 3 h in a shaking incubator at 180 rpm. After 3 h of growth, the cells were pelleted by centrifugation and suspended in 300 µL of 10 mM MgSO_4_ in PBS and 20 µL of 2 mg/mL lysostaphin. The samples were incubated for 1 h at 37°C with periodic vortexing. 300 µL of 2% IGEPAL in PBS and 20 µL of 20 mM PMSF were added to each sample. The tubes were incubated for 15 min on ice and then sonicated for 10 sec on ice with the probe sonicator on a setting pulse 10 sec and amplitude 50%. The cellular debris was pelleted by centrifugation at 20,817 rcf for 10 min. The supernatant was transferred into a clean tube, and total protein was quantified from the supernatants using BCA protein assay kit. 500 µL of supernatant containing 1 mg/mL protein was transferred into microcentrifuge tubes and sent to the IDeA National Resource for Quantitative Proteomics (Little Rock, AR) for proteomic analysis.

Total protein from each sample was reduced, alkylated, and purified by chloroform/methanol extraction prior to digestion with sequencing-grade modified porcine trypsin (Promega). Tryptic peptides were trapped and eluted on 3.5 µm CSH C_18_ resin (Waters) (4 mm × 75 µm) then separated by reverse phase XSelect CSH C_18_ 2.5 µm resin (Waters) on an in-line 150 × 75 µm column using an UltiMate 3000 RSLCnano system (Thermo). Mobile phase A was 0.1% formic acid and 0.5% acetonitrile in water, and mobile phase B was 0.1% formic acid in acetonitrile. The flow rate for the analysis was 0.3 µL/min. The peptides were eluted with a gradient that ramped from 2% mobile phase B to 5% over 2 min, then to 20% over 37 min, then to 40% over 9 min, then to 90% over 1 min. The column was washed at 90% mobile phase B for 4 min, then equilibrated to the starting condition of 2% for 7 min. Eluted peptides were ionized by electrospray (2.4 kV) through a heated capillary (275°C), followed by data collection on an Orbitrap Exploris 480 mass spectrometer (Thermo Scientific).

Precursor spectra were acquired with a scan from 385 to 1,015 *m/z* at a resolution set to 60,000 with 100% automatic gain control (AGC), max time of 50 msec, and an RF parameter at 40%. Data-independent acquisition (DIA) was configured on the Orbitrap 480 to acquire 50 × 12 *m/z* isolation windows at 15,000 resolution, normalized AGC target 500%, maximum injection time of 40 ms. A second DIA was acquired in a staggered window (12 *m/z*) pattern with optimized window placements.

Following data acquisition, data were searched using Spectronaut (Biognosys version 18.5) against the UniProt *Staphylococcus aureus* USA300 LAC database (April 2023) using the directDIA method with an identification precursor and protein q-value cutoff of 1%, generate decoys set to true, the protein inference workflow set to maxLFQ, inference algorithm set to IDPicker, quantity level set to MS2, cross-run normalization set to false, and the protein grouping quantification set to median peptide and precursor quantity (71). Protein MS2 intensity values were assessed for quality using ProteiNorm (72). The data were normalized using VSN (73) and analyzed using proteoDA to perform statistical analysis using Linear Models for Microarray Data (limma) with empirical Bayes (eBayes) smoothing to the standard errors (74–76). Proteins with a false discovery rate adjusted *P*-value < 0.05 and a fold change >2 were considered significant.

### Cytoplasmic pH measurement

BCECF-AM (ThermoFisher, B1150) dye was used to measure the cytoplasmic pH in *S. aureus* (31). Single colonies of *S. aureus* JE2 and Δ*menD* were inoculated into aeration culture tubes containing 5 mL of TSB and grown at 37°C for 24 h in a shaking incubator. Overnight cultures of JE2 and Δ*menD* were diluted 100- and 10-fold, respectively, in aeration culture tubes containing 5 mL of TSB supplemented with 0 or 18 mM sodium pyruvate. The bacterial cultures were grown for 4.5 h at 37°C in a shaking incubator, and then the cells were pelleted by centrifugation. The cell pellets were suspended in 1 mL of PBS. The OD_600_ of each sample was adjusted to 1. BCECF-AM dye was added to 250 µL of bacterial cells to a final concentration of 25 µM, and all samples were incubated for 30 min at 30°C in the dark. The cells were pelleted by centrifugation for 3 min at 20817 rcf, followed by suspension in 250 µL of PBS containing 0 or 25 mM sodium bicarbonate and 0 or 18 mM sodium pyruvate. 200 µL of cells from each sample tube was immediately transferred to a black-walled, 96-well plate (Corning), and fluorescence absorbance was measured at the excitation/emission wavelengths of 488/530 nm and 440/530 nm every 10 min for 1 h at 25°C with linear shaking at frequency 567 cpm (3 mm) in a Cytation5 plate reader (BioTek, Winooski, VT). A pH standard curve was generated at pH 6, 7, 8, 9, and 10 to calculate the cytoplasmic pH of bacterial cells. The MES buffer was used to make the pH 6 standard solution. Tris buffer was used to make the pH 7 and 8 standard solutions. CHES buffer was used to make the pH 9 and 10 standard solutions. 100 µM CCCP was added in each pH standard tube to permeabilize the cell membrane and equilibrate the internal and external pH levels, enabling the generation of a pH standard curve at pH 6, 7, 8, 9, and 10.

### Aminoglycoside antibiotic susceptibility assay

Overnight cultures of JE2 and SCV (Δ*menD*, *pbgS::Tn*, and *qoxB::Tn* Δ*cydB*) strains were diluted 100 and 10-fold, respectively, into 5 mL of TSB and grown shaking at 37°C for 5 h. The OD_600_ of the bacterial cells was adjusted to 0.6. A sterile cotton swab was dipped into each bacterial suspension and used to evenly spread the cells onto TSA plates buffered with 100 mM HEPES (pH 7.30), supplemented with 14 mM glucose, and containing either 0 or 25 mM sodium bicarbonate. The gentamicin MIC strip (Liofilchem S.r.l, Italy, 0.016–256 µg/mL) was placed in the middle of the plate using sterile forceps. The JE2 plates were incubated at 37°C for 24 h, and Δ*menD*, *pbgS::Tn*, and *qoxB::Tn* Δ*cydB* plates were incubated at 37°C for 48 h.

 Overnight cultures of JE2 and SCV (Δ*menD*, *pbgS::Tn*, and *qoxB::Tn* Δ*cydB*) strains were diluted 100 and 10-fold, respectively, into 5 mL of TSB and grown shaking at 37°C until the OD_600_ reached 0.6. The aminoglycoside susceptibility assays were performed on TSA buffered with 100 mM HEPES (pH 7.30) and supplemented with 14 mM glucose. For the gentamicin sensitivity assay, TSA plates were prepared with or without 25 mM sodium bicarbonate and supplemented with 1, 2.5, 5, or 10 µg/mL of gentamicin. For the tobramycin sensitivity assay, TSA plates were prepared with or without 25 mM sodium bicarbonate and supplemented with 0, 1, 5, 7.5, 10, or 12.5 µg/mL of tobramycin. Both gentamicin and tobramycin plates were spotted with 5 µL of bacterial cells serially diluted in 10-fold intervals from 10^0^ to 10^−4^ (77). All plates were incubated at 37°C, and bacterial growth on the plates was quantified after 24 h for JE2 and after 36 h for all SCVs.

 Overnight cultures of *S. aureus* Δ*menD* were diluted 10-fold into 5 mL of TSB and grown to an OD_600_ of 0.6 at 37°C in a shaking incubator. 10 µL of each outgrowth was added to 96-well plates containing 90 µL of PBS, then serially diluted in 10-fold intervals from 10^0^ to 10^−7^. 10 µL of each dilution was spotted onto TSA plates buffered with 100 mM HEPES (pH 7.3) and containing 14 mM glucose, 0 or 25 mM sodium bicarbonate, and 0 or 7.5 µg/ml gentamicin, or 0 or 15 µg/ml tobramycin. All plates were incubated at 37°C until visible colonies could be counted for CFU enumeration.

### *S. aureus* JE2 growth with bicarbonate and HOCl

HOCl sensitivity was determined as previously described (78). 10 µL of the *S. aureus* JE2 overnights were added to 50 µL of PBS containing 0 or 200 µM sodium hypochlorite (Ultra Bleach, Pure Bright) in a 96-well plate and incubated at room temperature for 40 min. After incubation, 60 mL of 2× TSB + HEPES media containing 0 or 100 mM sodium bicarbonate was added to each well. Bacterial growth was measured at 600 nm in an EPOCH 2 (BioTek, Winooski, VT) plate reader with readings taken every 30 min for 24 h at 37°C with linear shaking at 567 cpm (3 mm) (15).

### Neutrophil isolation and bacterial infection

Primary mouse neutrophils were purified from the bone marrow of wild-type C57BL/6J mice (79). Bone marrow was extracted from mouse femurs and tibias and mechanically dissociated into single-cell suspensions using a 70 µm strainer. Cells were collected by centrifugation and resuspended in MojoSort buffer (BioLegend, Cat. 480017). Neutrophils were purified by MojoSort mouse neutrophil isolation kit according to the manufacturer’s protocol (BioLegend, Cat. 480058). Cells were collected by centrifugation and resuspended in PBS. Cells were counted by hemocytometry, and the purity of neutrophils (>95%) was assessed by cytospin and Diff-Quick staining (Mercedes Scientific, Cat. MER 1002). *S. aureus* JE2 overnights were diluted 100-fold into 5 mL of TSB + HEPES media containing 0, 25, 50, or 100 mM sodium bicarbonate. The cultures were grown for 5 h at 37°C in a shaking incubator, then washed with PBS, and corrected to an OD_600_ of 1. The RPMI 1640 liquid media (Cytiva, Cat. SH30027.01) containing 24 mM sodium bicarbonate was used for the neutrophil assay. Neutrophils were adhered onto poly-L-lysine-coated 24-well plates (Sigma-Aldrich, Cat. P8920) and infected with *S. aureus* at a multiplicity of infection of 10. The infection was synchronized by centrifuging the plates at 1,500 rpm for 5 min followed by incubation for 10 min to allow the neutrophils to complete phagocytosis. Extracellular bacteria were killed by adding 10 U/mL of lysostaphin (Sigma Aldrich, Cat. L7386). At 2 h post-infection, the cells were washed with PBS and lysed with 1 mL of water +0.1% NP-40. Cell lysates were serially diluted in 10-fold intervals in PBS and plated onto TSA plates. Following incubation at 37°C for 24 h at 37°C, colonies were enumerated to quantify bacterial intracellular survival.

### Data presentation and statistical analyses

All experiments were repeated at least three times to ensure reproducibility. Replicate numbers are indicated for each experiment in the figure legend. All graphing and statistical analyses were performed in GraphPad Prism 10 (GraphPad Software, Boston, MA). GraphPad Prism was also used for statistical analysis and calculation of statistical significance by one-way ANOVA (nonparametric or mixed) followed by Tukey’s test. Specific statistical parameters for each individual experiment are indicated in the figure legends.

## SUPPLEMENTAL MATERIAL

Supplemental material spectrum.01685-25-s0001.docx Table S1; Fig. S1 to S7.

ASM does not own the copyrights to Supplemental Material that may be linked to, or accessed through, an article. The authors have granted ASM a non-exclusive, world-wide license to publish the Supplemental Material files. Please contact the corresponding author directly for reuse.
