# Supplementary material for: Defects in anaplerotic metabolism sensitize Staphylococcus aureus small colony variants to bicarbonate
Source: Microbiol Spectr. 2025 Sep 17;13(10):e01685-25. doi: 10.1128/spectrum.01685-25 (PMC12502694; doi:10.1128/spectrum.01685-25)
Supplement: Supplemental material — Table S1; Fig. S1 to S7. [file spectrum.01685-25-s0001.docx]

**Supplemental Material**

**Defects in anaplerotic metabolism sensitizes *Staphylococcus aureus* small colony variants to bicarbonate**

Asif Iqbal^a^, Zannatul H. Tumpa^a^, Wyatt W. Wittliff^a,b^, Bennett J. Blank^a^, Basel H. Abuaita^a^, William N. Beavers^a,b,#^

﻿^a^Department of Pathobiological Sciences, Louisiana State University and Agricultural and Mechanical College, School of Veterinary Medicine, Baton Rouge, Louisiana, USA

^b^Louisiana State University School of Veterinary Medicine Mass Spectrometry Resource Center, Louisiana State University and Agricultural and Mechanical College, School of Veterinary Medicine, Baton Rouge, Louisiana, USA

^#^Address correspondence to:

William N. Beavers, Ph.D.

LSU School of Veterinary Medicine

Department of Pathobiological Sciences

191 Skip Bertman Drive, Room 3315

Baton Rouge, LA 70803

Email: wbeavers@lsu.edu

**Table S1. list of primers used in the study.**

| **Primer name** | **Primer sequence** |
| --- | --- |
| menD-up_Fw | ATGTAATACGACTCACTATGATGAATGGCGAGAGTTTG |
| menD-up_Rv | TGCTTCATAAAATTCATATCGACTCCTAATGCATTC |
| menD-dw_Fw | CGATATGAATTTTATGAAGCAAACGTTGAG |
| menD-dw_Rv | CTTAAGCTCGGGCCCGAACAGAATGCTAAATCACCTTC |
| WNB00047 | ATAGTGAGTCGTATTACATGGTC |
| WNB00048 | GGGCCCGAGCTTAAGACTG |
| WNB00058 | CACTAACCTGCCCCGTTAGTTG |
| WNB00059 | ACACTTTATGCTTCCGGCTCG |
| menD-KO_Fw | ATCATCTTCGAGTTGTTGGAGGG |
| menD-KO_Rv | GTACATTACCGCCACCTACAG |
| qoxB::Tn_check_Fw | ATGAATTTTCCATGGGATCAATTACTAG |
| qoxB::Tn_check_Rv | TCATGACTCATGACTTACAGCCTC |
| cydB-up_Fw | CATGTAATACGACTCACTATAAAATAAATGGACACATTTCTTAATTAG |
| cydB-up_Rv | ﻿TCGTCTCAACTTTTCTATTTCTCCTCCTTGC |
| cydB-dw_Fw | ﻿AAATAGAAAAGTTGAGACGATACCCCAAC |
| cydB-dw_Rv | ﻿CCAGTCTTAAGCTCGGGCCCGAAAATGCGCTTGTAACAAG |
| cydB-KO_Fw | TGTATACATGTGCATACGTATTAAT |
| cydB-KO_Rv | AATTATTATAAATGAGTACCCCAAC |
| WNB00137 | ATGTTCACCTCAATTGTATTTATC |
| WNB00138 | GGATCCAAACAAGGGGGTAG |
| menD_Fw | AATACAATTGAGGTGAACATATGGGAAATCATAAAGCAG |
| menD_Rv | CTACCCCCTTGTTTGGATCCTTATAATGTGTCATGAATCATTTC |
| WNB00080 | CCAAAGCGCTAACCTTTTAGC |
| WNB00081 | GTTAGCTCACTCATTAGGCACC |
| Pyc::Tn_check_Fw | TTGAAACAAATAAAAAAGTTACTTGTTGCT |
| Pyc::Tn_check_Rv | TTAGTCAGTTGCTTTTTCAATTTCGATTAA |
| pyc_Fw | AATACAATTGAGGTGAACATATGAAACAAATAAAAAAGTTACTTG |
| pyc_Rv | CTACCCCCTTGTTTGGATCCTTAGTCAGTTGCTTTTTCAATTTC |
| cydB_Fw | AATACAATTGAGGTGAACATATGATTTATGCATTTATAGGTATAAC |
| cydB_Rv | CTACCCCCTTGTTTGGATCCTTATGATTTCTTTCCTTCAACATATTC |

**
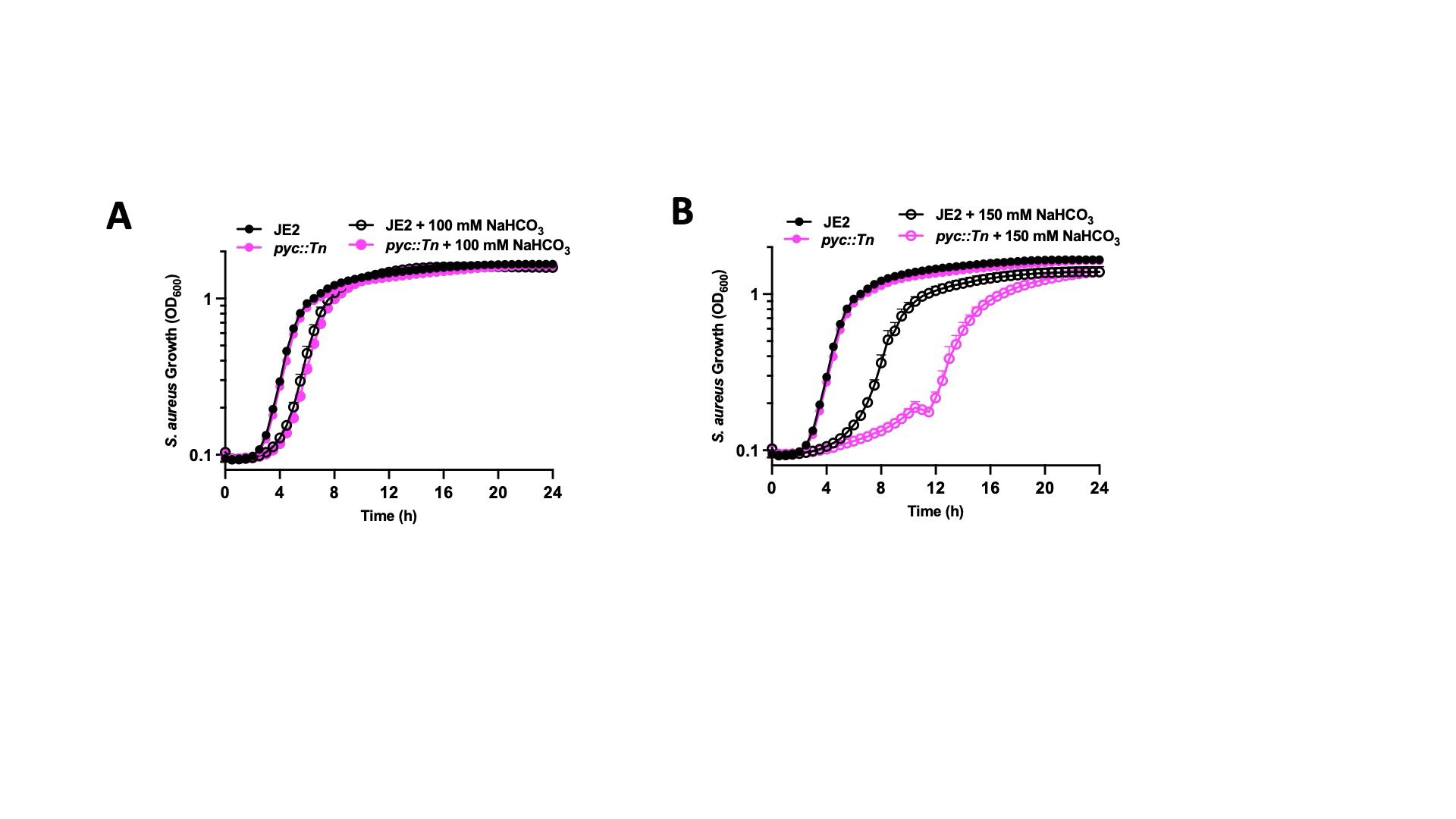
**

**Figure S1**: **Bicarbonate inhibits the growth of *S. aureus pyc::Tn***. (**A**) The growth of JE2 and *pyc::Tn* +/- 100 mM NaHCO_3_ was measured by monitoring the OD_600_ every 30 min for 24 h. Data are presented as mean +/- SEM of four biological replicates. (**B**) The growth of JE2 and *pyc::Tn* +/- 150 mM NaHCO_3_ was measured by monitoring the OD_600_ every 30 min for 24 h. Data are presented as mean +/- SEM of four biological replicates.


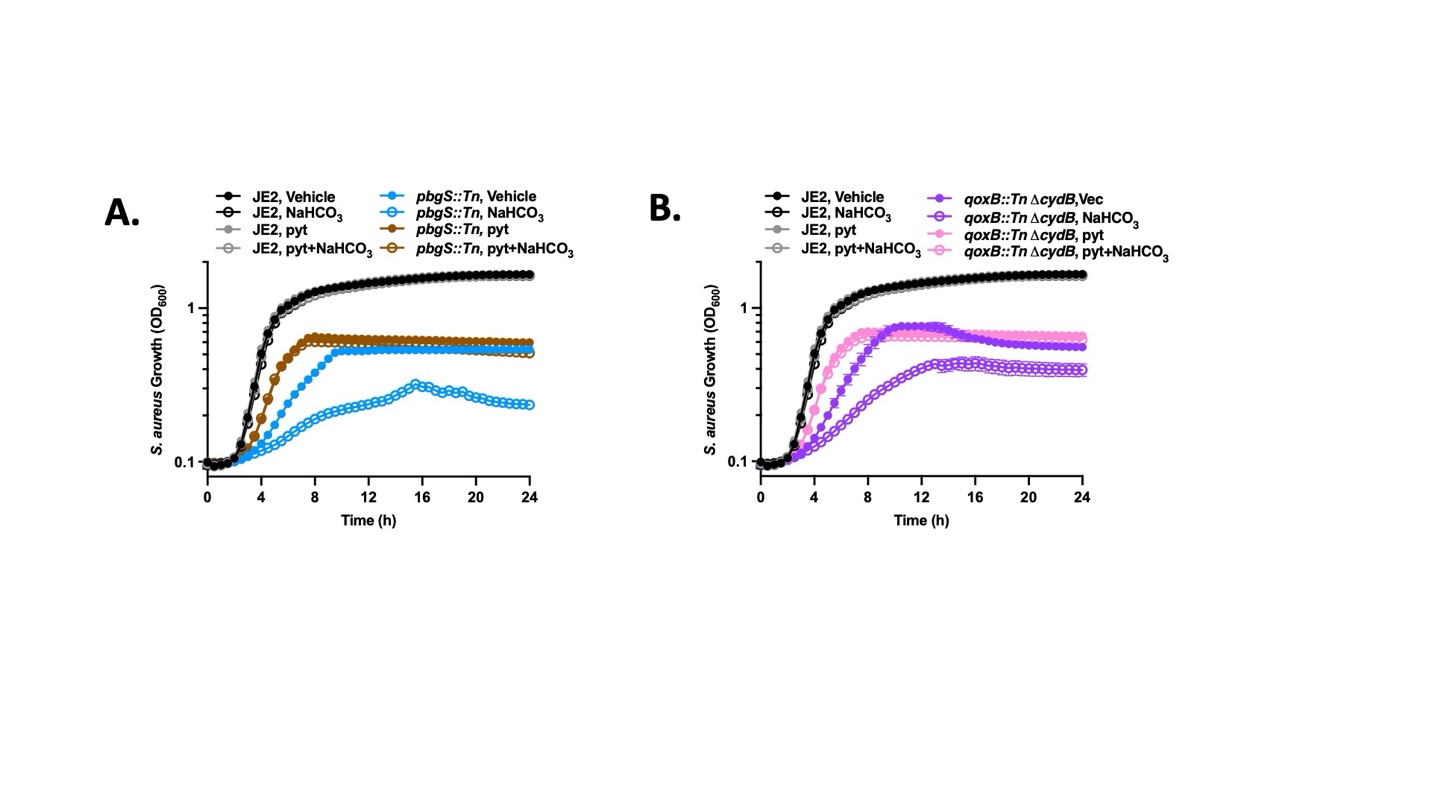


**Figure S2: Pyruvate protects against bicarbonate toxicity in *S. aureus* SCVs**. (**A**) The growth of *S. aureus* JE2 and *pbgS::Tn* was measured by monitoring the OD_600_ at 30 min intervals for 24 h +/- 50 mM NaHCO_3_ +/- 4.5 mM pyruvate (pyt). Data are presented as mean +/- SEM of three biological replicates. (**B**) The growth of *S. aureus* JE2 and *qoxB::Tn* △*cydB* was measured by monitoring the OD_600_ at 30 min intervals for 24 h +/- 50 mM NaHCO_3_ +/- 4.5 mM pyruvate (pyt). Data are presented as mean +/- SEM of three biological replicates.


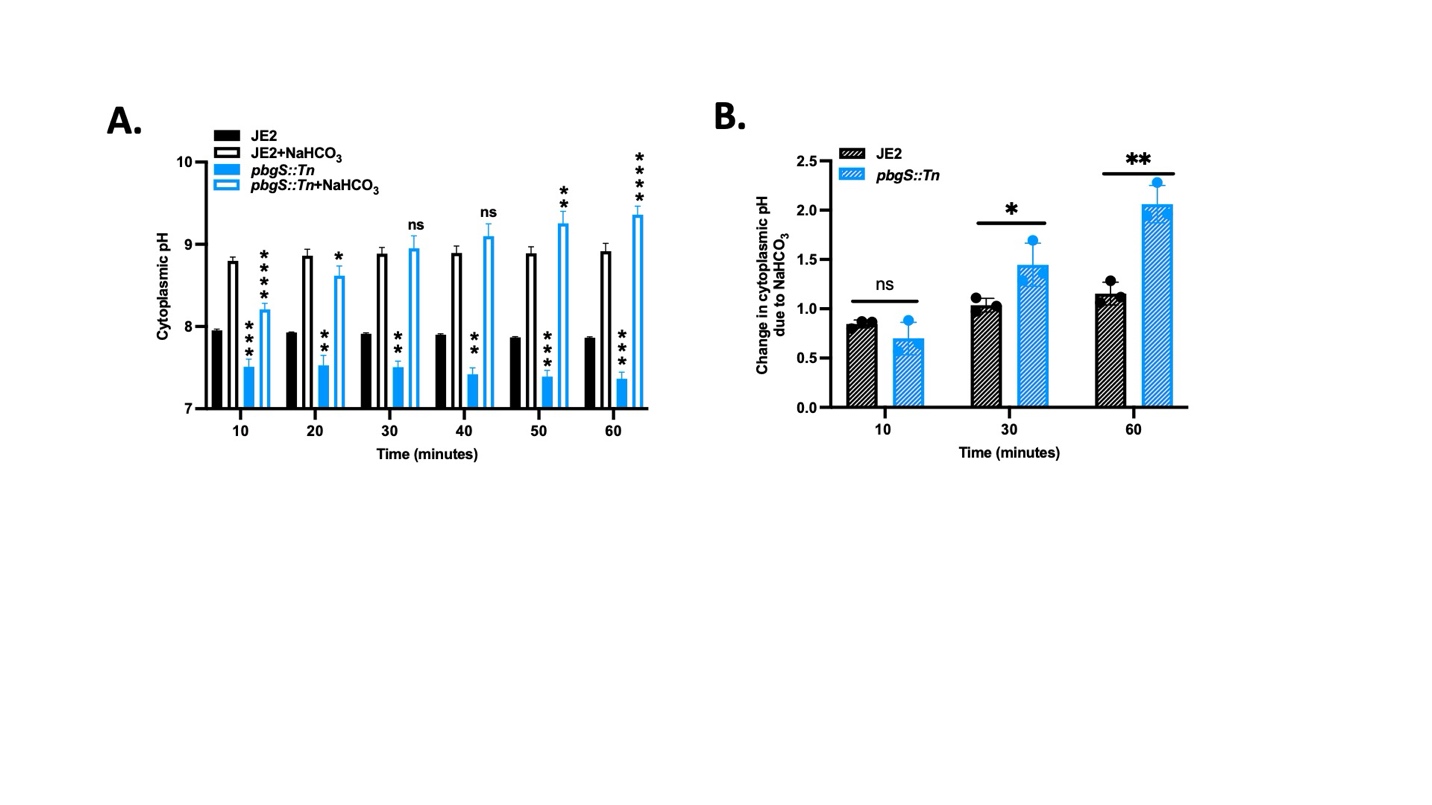

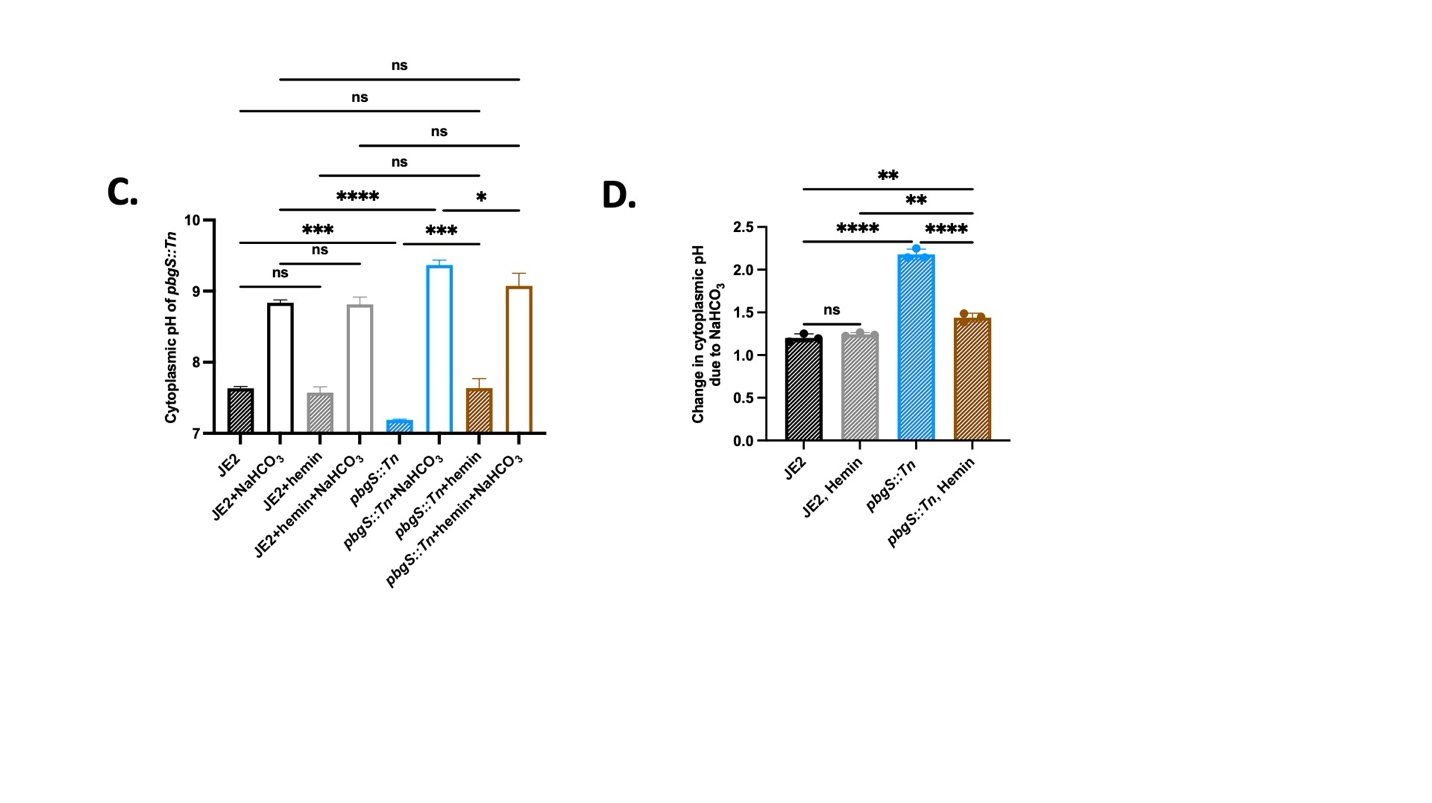


**Figure S3. Defective bicarbonate anaplerotic metabolism drives increased cytoplasmic alkalinization in *S. aureus* *pbgS::Tn***. (**A**) The cytoplasmic pH of JE2 and *pbgS::Tn* +/- 25 mM NaHCO_3_ was measured using BCECF-AM dye. Data are presented as mean +/- SD of three biological replicates. One-way ANOVA was used to determine statistical significance. The statistical comparisons shown compare JE2 to *pbgS::Tn* (closed bar) and JE2 + bicarbonate to *pbgS::Tn* + bicarbonate (open bars) at each time point where ns, *P* > 0.05; *, *P* < 0.05; **, *P* < 0.01; ***, *P* < 0.001; ****, *P* < 0.0001. (**B**) The change in cytoplasmic pH from panel A was calculated for each strain at different time points to determine how the pH changes following 25 mM NaHCO_3_ treatment. Data are presented as mean +/- SD of three biological replicates. One-way ANOVA was used to determine statistical significance where ns, *P* > 0.05; *, *P* < 0.05; **, *P* < 0.01. (**C**) The cytoplasmic pH of JE2 and *pbgS::Tn +/-* 5 μM hemin chloride +/- 25 mM NaHCO_3_ was measured following 60 min of growth using BCECF-AM dye. Data are presented as mean +/- SD of three biological replicates. One-way ANOVA was used to determine statistical significance where ns, *P* > 0.05; *, *P* < 0.05; ***, *P* < 0.001; ****, *P* < 0.000. (**D**) The change in cytoplasmic pH from panel C was calculated for each strain to determine how the pH changes following chemical complementation and 25 mM NaHCO_3_ treatment. Data are presented as mean +/- SD of three biological replicates. One-way ANOVA was used to determine statistical significance where ns, *P* > 0.05; **, *P* < 0.01; ****, *P* < 0.0001.

**
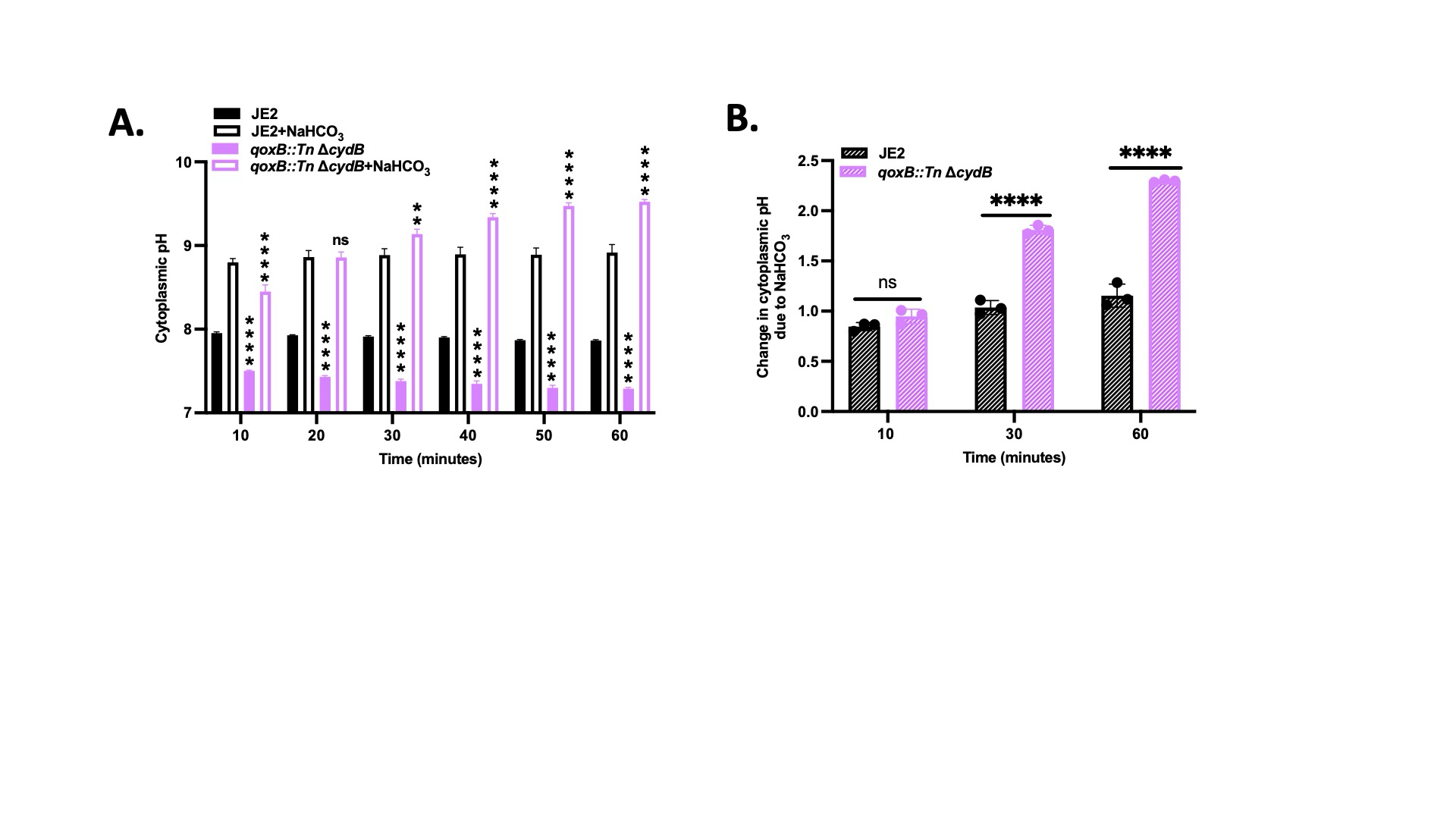
** **
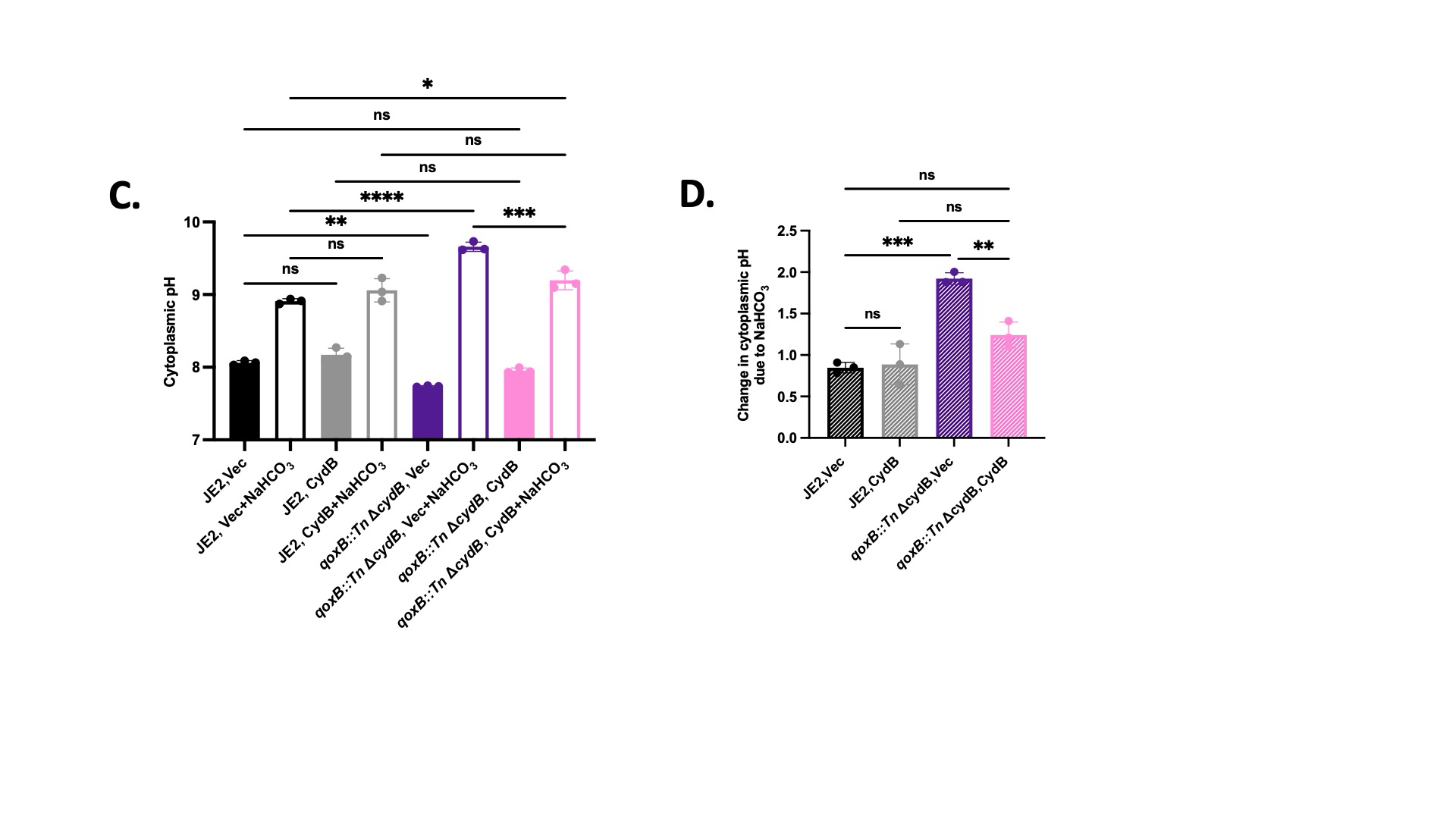
**

**Figure S4. Defective bicarbonate anaplerotic metabolism drives increased cytoplasmic alkalinization in *S. aureus qoxB::Tn* Δ*cydB***. (**A**) The cytoplasmic pH of JE2 and *qoxB::Tn* Δ*cydB* +/- 25 mM NaHCO_3_ was measured using BCECF-AM dye. Data are presented as mean +/- SD of three biological replicates. One-way ANOVA was used to determine statistical significance. The statistical comparisons shown compare JE2 to *qoxB::Tn* Δ*cydB* (closed bars) and JE2 + bicarbonate to *qoxB::Tn* Δ*cydB* + bicarbonate (open bars) at each time point where ns, *P* > 0.05; *, *P* < 0.05; **, *P* < 0.01; ***, *P* < 0.001; ****, *P* < 0.0001. (**B**) The change in cytoplasmic pH from panel A was calculated for each strain at different time points to determine how the pH changes following 25 mM NaHCO_3_ treatment. Data are presented as mean +/- SD of three biological replicates. One-way ANOVA was used to determine statistical significance where ns, *P* > 0.05; ****, *P* < 0.000. (**C**) The cytoplasmic pH of JE2 and *qoxB::Tn* Δ*cydB* transformed with empty control vector pOS1.P*_Igt_* (Vec) or pOS1.P*_Igt_.cydB* (CydB) +/- 25 mM NaHCO_3_ was measured following 60 min of growth using BCECF-AM dye. Data are presented as mean +/- SD of three biological replicates. One-way ANOVA was used to determine statistical significance where ns, *P* > 0.05; *, *P* < 0.05; **, *P* < 0.01; ***, *P* < 0.001; ****, *P* < 0.000. (**D**) The change in cytoplasmic pH from panel C was calculated for each strain to determine how the pH changes following 25 mM NaHCO_3_ treatment. Data are presented as mean +/- SD of three biological replicates. One-way ANOVA was used to determine statistical significance where ns, *P* > 0.05; **, *P* < 0.01; ***, *P* < 0.001.


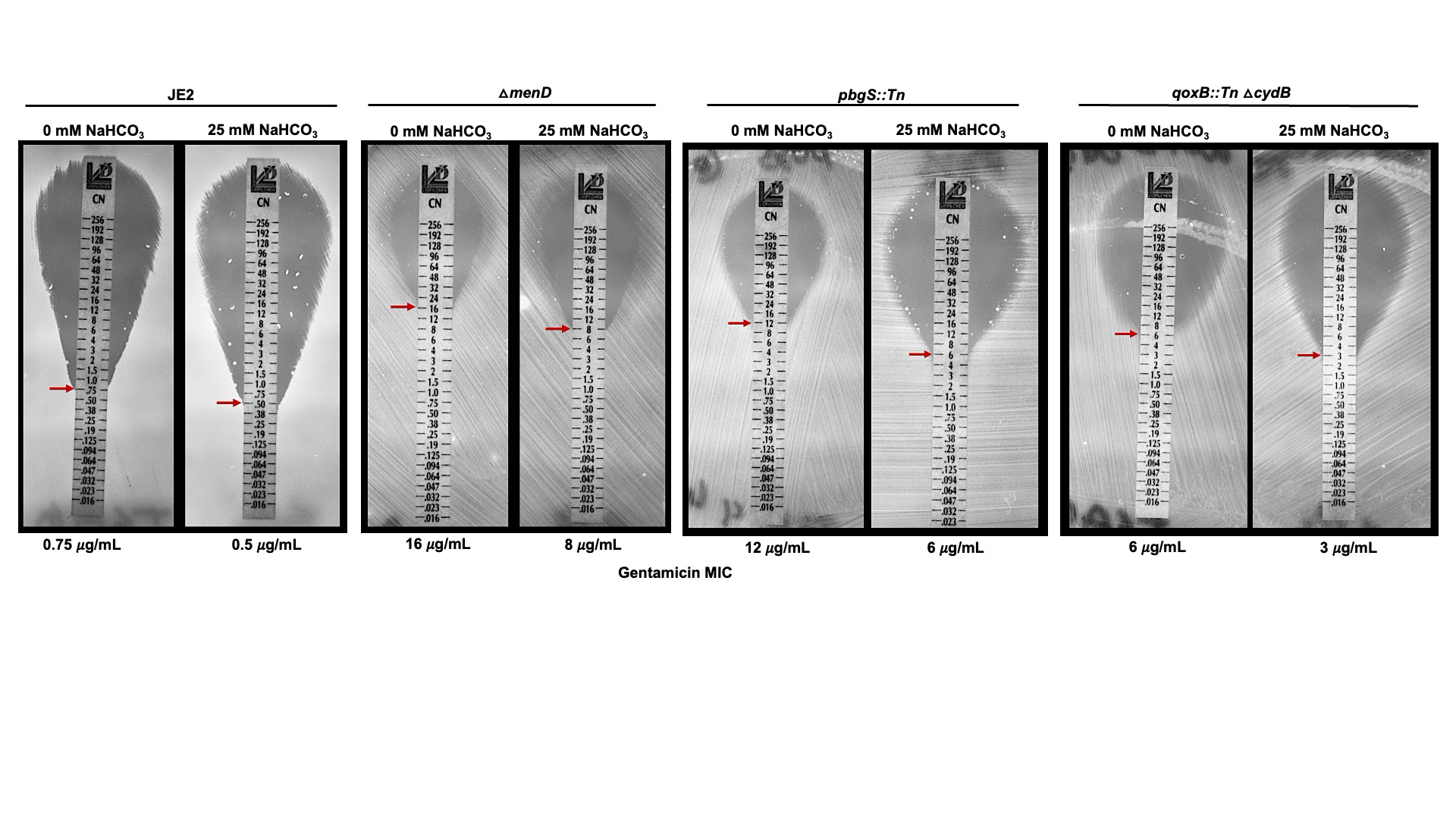


**Figure S5. Gentamicin MIC (minimum inhibitory concentration) determination for *S. aureus* JE2, Δ*menD*, *pbgS::Tn*, and *qoxB::Tn* Δ*cydB***. The overnight cultures of bacterial strains were grown for 5 h, and their OD_600_ were adjusted to 0.6. The bacterial cells were spread onto TSA + 14 mM glucose -/+ 25 mM sodium bicarbonate (NaHCO_3_). JE2 plates were incubated to 24 h at 37 °C. Δ*menD*, *pbgS::Tn*, and *qoxB::Tn* Δ*cydB* for 48 h at 37 °C.

**
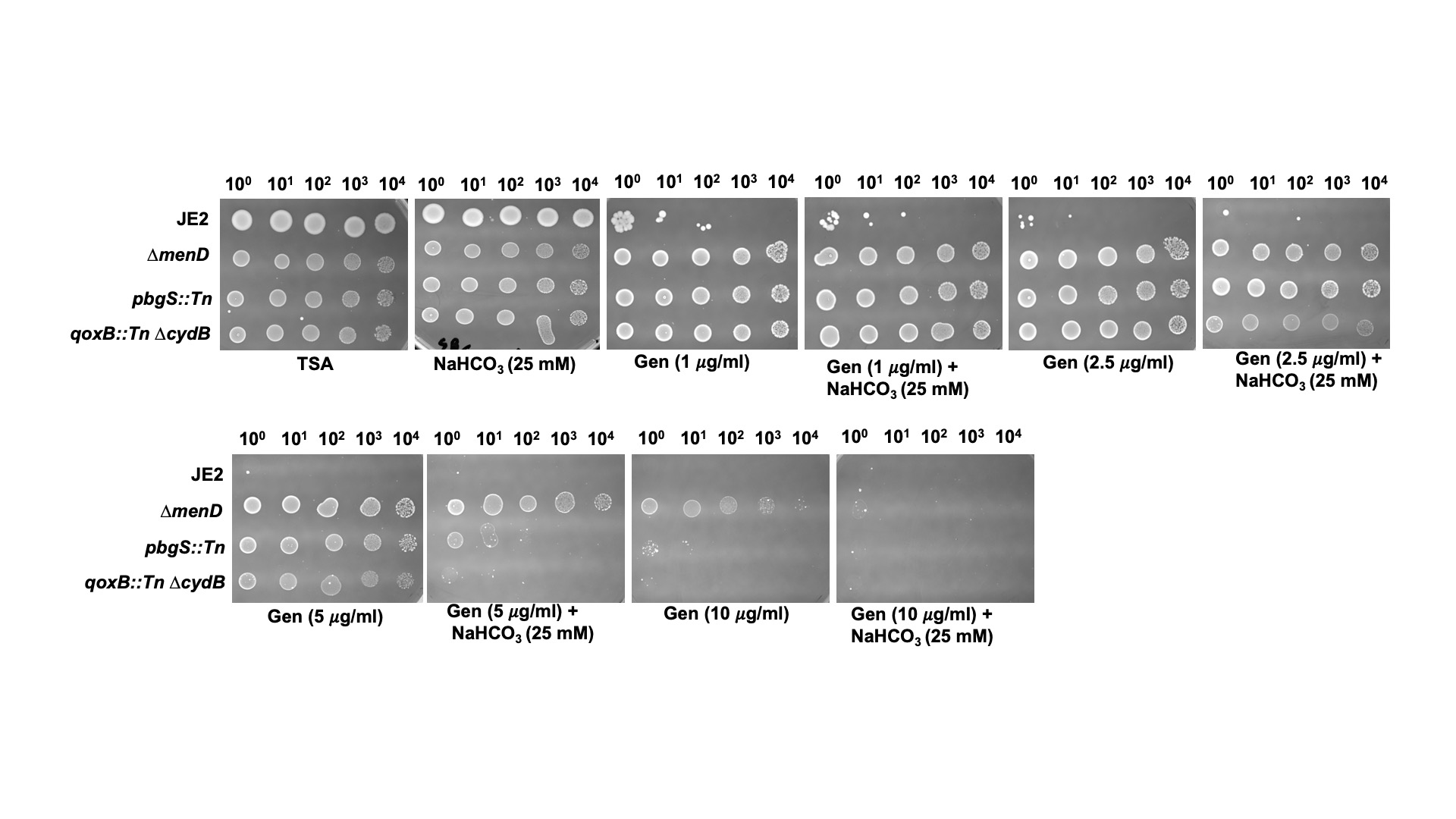
**

**Figure S6: Bicarbonate restores gentamicin sensitivity in *S. aureus* SCVs.** Serially diluted JE2, △*menD, pbgS::Tn,* and *qoxB::Tn* △*cydB* were spotted and grown on TSA containing 14 mM glucose, +/- 25 mM sodium bicarbonate (NaHCO_3_), and +/- 1, 2.5, 5, or 10 μg/mL gentamicin (Gen).

**
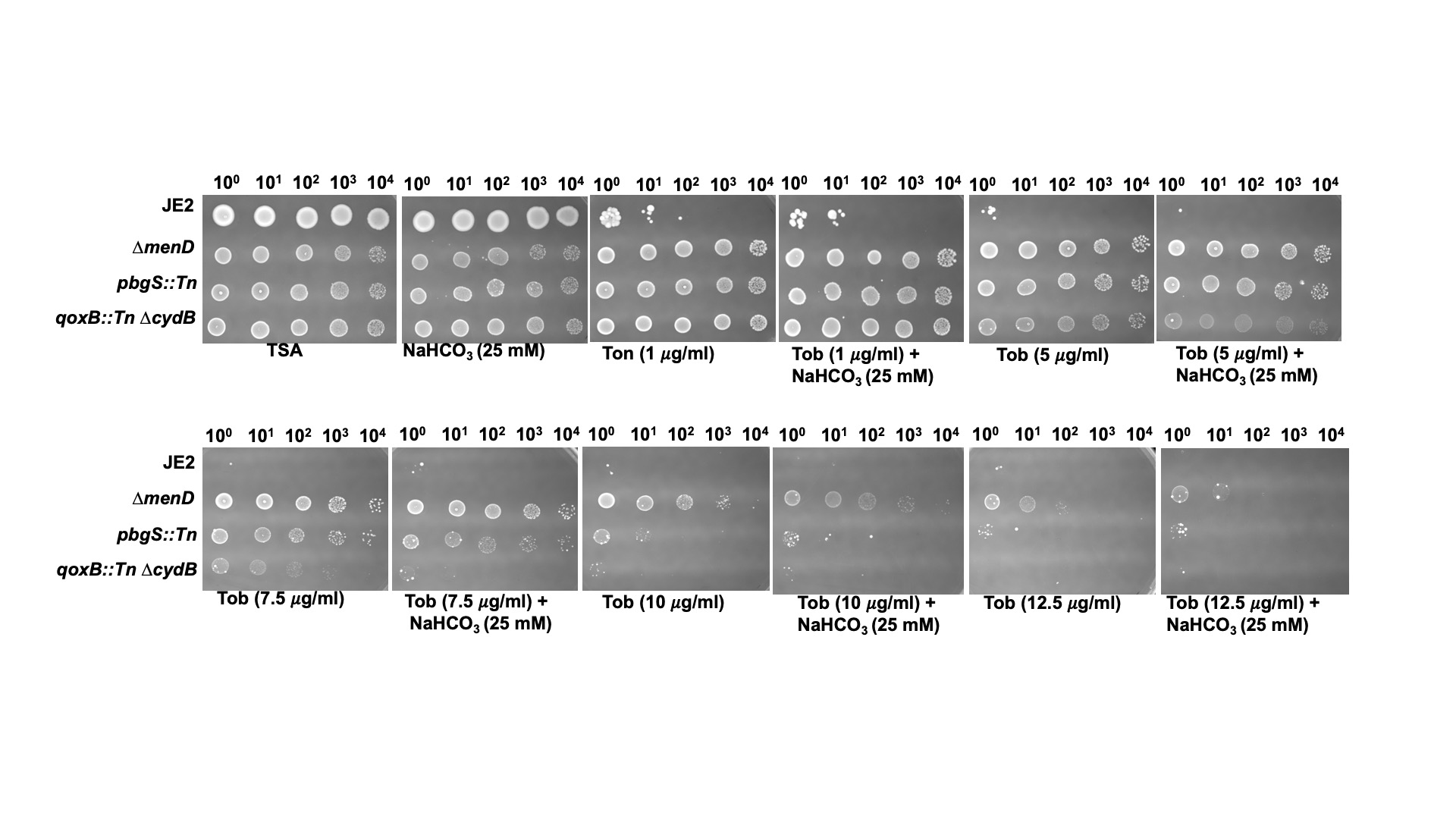
**

**Figure S7: Bicarbonate restores tobramycin sensitivity in *S. aureus* SCVs.** Serially diluted JE2, △*menD, pbgS::Tn,* and *qoxB::Tn* △*cydB* were spotted and grown on TSA containing 14 mM glucose, +/- 25 mM sodium bicarbonate (NaHCO_3_), and +/- 1, 5, 7.5, 10, or 12,5 μg/ml tobramycin (Tob).
